# Multiblock Integration and Modeling of Localized Microbiome, Metabolome, and Clinical Metadata to Identify Biomarkers Predictive of Outcome in Veterans with Non-Healing Wounds

**DOI:** 10.1101/2025.09.07.674782

**Published:** 2025-09-10

**Authors:** Catherine B. Anders, Hannah Smith, Jeremy Boyd, Michael C. Davis, Tyler M.W. Lawton, Matthew Fields, Chiachi Hwang, Margaret M. Doucette, Mary Cloud B. Ammons

**Affiliations:** 1 Research Service, Boise VA Medical Center (BVAMC), Boise, ID 83702; 2 Idaho Veterans Research & Education Foundation (IVREF), Boise VA Medical Center (BVAMC), Boise, ID 83702; 3 Department of Physical Medicine & Rehabilitation, Boise VA Medical Center (BVAMC), Boise, ID 83702; 4 Center for Biofilm Engineering, Montana State University, Bozeman, MT 59717

## Abstract

**Background:**

Type 2 Diabetes affects more than 37 million people in the United States and is the number one cause of lower-limb amputation in adults due to diabetic foot ulcers (DFU). The chronic wound microenvironment consists of a complex milieu of host cells, microbial species, and metabolites. While much is known about the wound microbiome, our knowledge of the metabolic landscape and its influence on microbial diversity and wound healing is limited. Furthermore, the integration of these complex datasets into a predictive model with relevance to clinical outcome is almost non-existent. Here, we present a multiomics data analysis coupled with machine-learning cross validation of microbiome and metabolome profiles from human chronic wounds. The model was integrated with patient metadata to determine predictive correlation to clinical outcome under standard of care.

**Methods:**

Microbial ribosomal RNA (rRNA) and total metabolites were extracted from 45 DFU debridement samples collected from 13 patients at the Boise VA Medical Center. Of 45 samples analyzed, 25 samples were isolated from wounds that failed to respond to standard treatment while the remaining 20 samples were taken from wounds that progressed to healing and remained closed for >30 days. 16S rRNA sequencing and global metabolomics were performed and clinical metadata was collected from patient records. Healing outcome was modeled as a function of three blocks of features (N = 21 clinical, 634 microbiome, and 865 metabolome) using DIABLO (**D**ata **I**ntegration **A**nalysis for **B**iomarker Discovery using **L**atent C**o**mponents) based on multiblock sparse partial least squares discriminant analysis (sPLS-DA) which performs feature selection using LASSO regularization. Seven-fold cross-validation with 100 repeats was used to find the amount of regularization associated with the smallest predictive error.

**Results:**

The final model selected a total of 176 features (N = 15 clinical, 8 microbiome, and 153 metabolome) and was able to predict the clinical outcome with an overall error rate of 6.44%.

**Conclusion:**

These results indicate that the integration of wound microbiome and metabolomics data with patient clinical metadata can be utilized to predict clinical outcomes regarding wound healing and with low error rates. Furthermore, the biomarkers selected within the model may offer novel insights into wound microenvironment composition, reveal innovative therapeutic approaches, and improve treatment efficacy in difficult to heal wounds.

## INTRODUCTION

The number of people over the age of 18 diagnosed with diabetes mellitus (DM) quadrupled from 5.5 million to 21.9 million from 1980 to 2014 representing 9.1% of the adult population (1). These numbers are projected to increase to 39.7 million (13.9 %) in 2030 and 60.6 million (17.9%) in 2060 (1). Within this affected population, it is estimated that up to 44% will develop a diabetic foot ulcer (DFU) infection (2) which leads to amputation in approximately 25% of this population due to failure to heal (3). Independent risk factors associated with the development of DFU infections and amputation are variable and include peripheral neuropathy, peripheral arterial disease (PAD), periwound edema, wound area, wound depth, elevated C-reactive protein, polymicrobial infections, extended spectrum beta-lactamase-producing Gram-negative bacterium, hospital admission for DFUs, and vancomycin treatment (3–6). Given that the 5-year mortality rates following lower extremity amputations are approximately 50% (7), a clearer understanding of the underlying factors impacting healing outcomes of DFUs is needed.

Bacterial bioburden, biofilm formation, and recurrent infections have been identified as contributing factors to the development of chronic wounds generally defined as wounds that persist 4 – 6 weeks post wounding and do not respond to standard treatment regimens (8). It is estimated that 50% of chronic DFUs will be infected upon examination but fail to demonstrate clinical signs of infection due to PAD or patient immune system dysregulation (9). While traditional wound culture methods employed by most diagnostic labs can identify the presence of pathogenic bacteria, this information may offer little diagnostic value as all open wounds will colonize bacteria and culture-based methods are limited to bacteria that will grow well in culture and often underestimate the true bacterial diversity compared to culture-independent methods (10,11). Previous studies focused on culture-based methods have identified *Staphylococcus aureus*, *Enterococcus faecalis*, *Enterobacter cloacae*, *Pseudomonas aeruginosa*, *Acinetobacter baumanni*, *Escherichia coli*, *Corynebacterium* species (spp.), *Klebsiella* spp., and *Streptococcus* spp. (4,12,13) as pathogens that contribute to the infections within the wound microenvironment. While culture-based methods can identify pathogenic bacteria that will grow well in culture, these methods are limited in their ability to detect less abundant, non-pathogenic bacteria and anaerobic species which could significantly influence healing (11). Culture-independent techniques, such as 16S ribosomal RNA (rRNA) and next generation sequencing (NGS), allow for a more comprehensive assessment of microbiota diversity that facilitate the use of complex statistical analyses capable of identifying predictors that influence healing progression, including the assessment of therapeutic treatment, subtle changes in microbial diversity over time, the presence of biofilm associated pathogens, and bacterium associated with patient metabolic status (12,14–18).

Within chronic wounds, colonizing bacteria exist in viable, dormant and non-viable physiological states with pathogenic bacterial species demonstrating high metabolic activity while metabolically dormant species contribute to chronic wound persistence (19). It has been hypothesized that concentration gradients of key metabolites established during bacterial colonization and biofilm formation drive the activation of these variable metabolic states (20); however, very few studies have sought to characterize the metabolome of chronic wounds. Ammons, et al. (2015) examined the microbiome and metabolome of chronic pressure ulcers and determined that distinct shifts in the metabolome of these wounds corresponded to changes in the microbiota present (19). Likewise, a temporal healing study of acute wounds revealed that decreases in *Staphylococcus aureus* corresponded to increases in *Propionibacterium* and collagen levels within the wounds (21). These studies indicate that it is particularly important to identify how the metabolome of the chronic wound microenvironment modulates the composition of the colonizing bacteria and how those shifts impact patient healing.

While the contribution of colonizing microbiota and localized metabolic environment are known to act synergistically with patient predisposition to drive clinical outcome in wound healing, to our knowledge, there are no studies that integrate this high-dimensional data into a single predictive model of clinical outcome. In this study, we utilize the emergent, leading method for handling integration of high-dimensional data: DIABLO (**D**ata **I**ntegration **A**nalysis for **B**iomarker Discovery using **L**atent C**o**mponents), based on sparse partial least squares discriminant analysis (sPLS-DA) to model the major predictors of healing and non-healing in patients with chronic wounds. This biomarker discovery approach enables integration of comprehensive microbiome and metabolome data with metadata extracted from the patient clinical record, as well as interrogation of the correlative relationship between biomarkers contributing to model robustness. This study addresses the need for the comprehensive characterization of both the microbiome and the metabolome of chronic wounds and correlates those findings to healing outcome and patient metadata. Our results demonstrate the usefulness of this multiomics statistical modelling approach, as well as providing a foundation for the novel development of clinically impactful wound healing diagnostics and therapeutics. Furthermore, this approach establishes the DIABLO integrative modeling approach as a basis for hypothesis generation regarding mechanisms of interaction between the wound metabolome, host systemic health, and the wound microbiome in non-healing wounds.

## METHODS (outlined in Figure 1)

### Ethics statement

Written informed consent was obtained from patients (ages 18–60 years old) being treated in the Wound Care Clinic at the Boise VA Medical Center (BVAMC) and enrolled in the study approved by the VA Puget Sound institutional review board (IRB, IRBNet ID:1647932). Donor demographics, data from electronic medical records, and serial wound debridement tissue discarded as part of patient standard of care was collected. After collection, samples were stored at −70°C until analysis.

### Patient Cohort

For this study, donor subjects were recruited from the BVAMC Wound Care Clinic and were selected based on the presence of a non-acute, non-healing wound, defined as being recalcitrant to healing for over 30 days. Exclusion criteria included < 18 years of age, pregnant, or nursing. Subjects were followed over the course of standard of care wound treatment and serial wound debridement were collected for later analysis. A total of 75 subjects were enrolled in the study with 25 subjects responding to standard of care (Responders) and 50 subjects not responding to standard of care (Non-Responders) within the course of treatment. For the purposes of this study, a Responder is any subject who had resolved a single or multiple wounds to >80% closure without the co-occurrence of another wound and remained healed for >30 days, whereas a Non-Responder is any subject who underwent amputation due to a failure to heal or presented with multiple wounds either concurrently or consecutively over an extended period without prolonged wound resolution or healing of all wounds for a period of >30 days. Demographic and clinical data from the subject medical record was extracted (Supplemental Table 1) to characterize the total patient cohort and a random selection of 13 subjects, 20 Responders and 25 Non-Responders, were selected to have their serial debridement tissue profiled for microbiome colonization and local metabolite landscape, resulting in a total of 48 wound debridement tissue samples characterized for microbiome and metabolome (Table 1).

### Metabolome

#### Intracellular Metabolite Isolation.

With the goal of creating a comprehensive snapshot of the chronic wound metabolome, 10–30 mg of wound debridement tissue was homogenized using four cycles of 120 seconds each at 6.0 m/s with a 300 second delay between cycles in FastPrep^®^ lysing matrix D tubes (MP Biomedicals; Auckland, New Zealand) utilizing the FastPrep-24^™^ 5G Homogenizer (MP Biomedicals; Auckland, New Zealand). Samples were refrozen between each homogenization cycle with liquid nitrogen. The resulting tissue homogenate was suspended 375 μL of ice-cold 100% methanol (Honeywell; Muskegon, MI, USA) and 375 μL of ice-cold ultrapure distilled water (Invitrogen; Grand Island NY, USA) followed by the addition of 700 μL of ice-cold chloroform (Acros Organics; Thermo Fisher Scientific; Waltham, MA, USA). The tubes were homogenized again using the same settings. The homogenized samples were centrifuged at 16,000 × g for 5 minutes at 4°C and placed immediately on ice. The polar (methanol/water) layer and non-polar (chloroform) layers were subsequently transferred to 1.5 mL protein low binding microcentrifuge tubes. These metabolite suspensions were lyophilized overnight without heat on a ThermoScientific^™^ Savant^™^ ISS110 SpeedVac^™^ (Waltham, MA, USA) and stored at −80°C until the samples were shipped to Metabolon for further analysis. The remaining interphase layer was flash-frozen in liquid nitrogen and stored at −80°C until RNA extraction.

#### Metabolite Detection, Identification, and Quantification.

All samples were analyzed by Metabolon (Morrisville, NC, USA) using four ultra-high-performance liquid chromatography/tandem accurate mass spectrometry (UHPLC/MS/MS) methods. The following is a summary of Metabolon’s procedure. All methods utilized a Waters ACQUITY ultra-performance liquid chromatography (UPLC) and a Thermo Scientific Q-Exactive high resolution/accurate mass spectrometer interfaced with a heated electrospray ionization (HESI-II) source and Orbitrap mass analyzer operated at 35,000 mass resolution. Lyophilized samples were reconstituted in compatible solvents containing a series of standards at fixed concentrations to assay consistency. Three aliquots were gradient eluted from a C18 column (Waters UPLC BEH C18–2.1×100 mm, 1.7 μm) using water and methanol, containing 0.05% perfluoropentanoic acid (PFPA) and 0.1% formic acid to detect hydrophilic compounds, methanol, acetonitrile, water, 0.05% PFPA and 0.01% formic acid for hydrophobic compounds, and methanol and water with 6.5 mM ammonium bicarbonate at pH 8 to for basic compounds. A fourth aliquot was analyzed via negative ionization following elution from a HILIC column (Waters UPLC BEH Amide 2.1×150 mm, 1.7 μm) using a gradient consisting of water and acetonitrile with 10mM ammonium formate, pH 10.8. The MS analysis alternated between MS and data-dependent MS scans using dynamic exclusion. The scan range varied slightly between methods but covered 70–1000 m/z. Controls included a pooled matrix sample that served as a technical replicate throughout the data set, extracted water samples as process blanks; and a cocktail of quality control (QC) standards.

Raw data was extracted, peak-identified and QC processed using Metabolon’s hardware and software. Compounds were identified by comparison to library entries of purified standards or recurrent unknown entities. Biochemical identifications are based on three criteria: retention index within a narrow RI window of the proposed identification, accurate mass match to the library +/− 10 ppm, and the MS/MS forward and reverse scores between the experimental data and authentic standards. The resulting data was then normalized to sample weight to account for differences in metabolite levels due to differences in the amount of material present in each sample. After normalization, peak intensity values were uploaded into MetaboAnalyst 4.0 (Ste. Anne de Bellevue, Quebec)(22), normalized using pareto scaling, statistically analyzed, and evaluated using the Enrichment Analysis and Pathway Analysis modules.

#### Metabolomics Data Deposition.

Global metabolomics data was deposited in the National Metabolomics Data Repository accessed through the Metabolomics Workbench (metabolomicsworkbench.org) and supported by the NIH Common Fund Metabolomics Program. Original scale metabolite value by raw area count and associated metadata will be released from embargo upon publication.

### Microbiome profiling of wound-colonizing bacteria

#### 16S rRNA and cDNA isolation.

16S rRNA was purified from the wound debridement samples utilizing the RNeasy PowerBiofilm Kit (Germantown, MD, USA). The lysing tubes containing the interphase layer from the metabolite extraction were resuspended in the supplied lysis buffer supplemented with 1% (v/v) of β-mercaptoethanol and homogenized, as described above. Following lysis, the RNA was extracted and purified according to the manufacturer’s instructions. The integrity of the extracted RNA was evaluated on an Agilent 2200 Tape Station (Santa Clara, CA, USA). cDNA was generated from the extracted 16s rRNA using the Applied Biosystems High-Capacity cDNA Reverse Transcription Kit with RNAse Inhibitor according to manufacturer’s instructions.

#### 16S rRNA sequencing.

The V1-V3 region of bacterial 16S rRNA gene was amplified from extracted tissue using Bac_8F and Bac_529r primers, Taq polymerase (Takara Biotechnology), and standard PCR cycling: 94°C for 30 secs followed by 30 cycles denaturation at 94°C for 30 secs, annealing at 52°C for 30 sec, and extension at 72°C for 30 seconds; followed by a final extension at 72°C for 10 mins with adaptor sequences for Illumina sequencing. The final library was pair-end sequenced (2 × 300 bp) on an Illumina MiSeq at the Center for Biofilm Engineering at Montana State University, using the MiSeq reagent kit v3, 600 cycles.

#### Taxonomic and abundance analysis of Sequence data.

The resulting sequence data were analyzed using QIIME2–2022.11 (22), running in Ubuntu 22.04.1. The DADA2 package (23) wrapped inside the QIIME2 pipeline was used for denoising and constructing amplicon sequence variants (ASVs). The range of reads per sample was 17545 to 335,965, with a median of 83,945, and a total of 6,464,128 reads for all samples. Reverse reads truncated at 257 bp to remove low quality (q<30) base calls. One sample with a high percentage of chimeric sequences was dropped from analysis. Forward and reverse reads were trimmed by 20 and 15 bp, respectively, to remove primer sequences. Low-quality reads and those that had fewer than 300 hits were also removed. Taxonomic analysis was performed using a pre-trained Bayesian naive classifier, (gg-13–8-99-nb-classifier). Classified bacterial taxa down to genus level were used for input into the statistical model. For insight into species distributions of identified pathogens, the gg99 classifier was used for species calls. Where species were not identified, a BLAST search of the sequences against the curated NCBI 16S/ITS database was performed. Hits with ≥ 99% identity, over the entire sequenced V1-V3 region, were considered adequate for identification. If several species were within the ≥ 99% range, a check into the relatedness of the species was performed, so that type of strain or species group could be identified. If no species or strain was ≥ 99% identical but the same species was identified from three or more database entries with ≥ 97%, that was also used to tentatively identify species.

Heat trees, phylogenetic trees with differential abundances shown with heat map colors, were constructed using the R package metacoder (24). Heat trees were generated using taxonomic information from the set of 634 taxa, to give an overview of the diversity and phylogenetic relationships among microbes identified in the samples.

### Multiblock, Integrative Modeling

Following clinical annotation of the collected samples and data collection, three distinct dataset blocks were compiled and herein referred to as the metabolome (metabolomics dataset), microbiome (16S rRNA sequencing data), and clinical markers (patient metadata). Samples within the compiled dataset were then classified according to patient wound healing status, specifically Responder and Non-Responder, as detailed above.

The following data preprocessing steps were taken prior to modeling: features with near zero variance were excluded from all datasets; features in the microbiome dataset with OTU counts less than 1% of the total were excluded and all remaining microbiome features underwent the centered log transformation (25); and features in all datasets were standardized to zero means and unit variances (21).

Clinical care outcome was modeled as a function of metabolome, microbiome, and clinical datasets using DIABLO (**D**ata **I**ntegration **A**nalysis for **B**iomarker Discovery using **L**atent C**o**mponents) (26–28). The single-dataset counterpart to DIABLO is sPLS-DA (sparse partial least squares discriminant analysis). Prior to fitting the DIABLO model, two-component sPLS-DA models were fit using each of the metabolome, microbiome, and clinical datasets on their own to predict the wound healing outcome. To determine the amount of regularization associated with the best predictive performance, models with different numbers of features were fit, with feature number values ranging from one to half the number available after preprocessing. The best model for each dataset was chosen using seven-fold cross-validation with 100 repeats. The single dataset results were used to inform construction of the DIABLO tuning grid. The DIABLO model was parameterized with between-datasets weights of 0.1 and two components per dataset. The weight values prioritize finding a model fit that discriminates between outcome classes but still learns inter-dataset correlations (28). The number of components was informed by single-dataset modeling (Supplemental Figure 1), which also indicated better predictive performance on the metabolome and microbiome datasets when a minimum of 80 and two features were selected, respectively. These values were incorporated into a grid representing possible numbers of features that could be selected onto the DIABLO components for each dataset. There were 18 possible metabolome values running from 80 to 420, 17 microbiome values from two to 90, and ten clinical values running from one to ten. The maximum number of features that could be selected for each dataset was always half the number of features available after preprocessing. This restriction enforced significant regularization and acted as a hedge against overfitting. In all, 6,120 candidate DIABLO models were fit, representing all possible combinations of selected numbers of features in each dataset, on each component. Seven-fold cross-validation with 100 repeats was used to identify the final model—i.e., the fit with the lowest predictive error.

## RESULTS

For this study, an integrative model was built to predict whether wounds heal under standard of care treatment based on metabolome, microbiome, and clinical datasets. A key feature of the model is that it performs feature selection. With model feature selection, the selected features were fed into non-integrative metabolome and microbiome analyses. The integrative model was in two ways. First, the model itself—the features selected from each dataset and the pattern of correlations among features—was used to generate systems-level hypotheses regarding why some wounds in the study population heal while others do not. Second, selected metabolome and microbiome features were fed into additional non-integrative analyses: metabolite set enrichment analysis (MSEA), and investigation of bacterial abundances by outcome class.

### Patient Cohort Characterization

For this study, the patient cohort was recruited from the Boise VA Wound Clinic and subjects were selected for having wounds recalcitrant to acute healing, as defined above (Supplemental Table 1). The average age for the cohort was between 70 – 80 years old, with average age increasing based metabolic status from diabetic to pre-diabetic to non-diabetic. While the average age of Responder and Non-Responder patients with diabetes is similar, the average age of the patients without diabetes who were Non-Responders is notably higher than the paired group of Responders. Overall, the donor cohort was predominantly prediabetic (40% of Responders, 12% of Non-Responders) and diabetic (40% of Responders, 80% of Non-Responders). Non-diabetic patients accounted for a minority of the Responder and Non-Responder cohorts (20% and 8%, respectively). Nearly the entire cohort was classified as overweight or obese based on Body Mass Index (BMI ≥ 25), with a statistically significant difference between the Responder and Non-Responder classification. In addition, the cohort was notably hyperglycemic with a statistically significant difference in blood glucose level between Responder and Non-Responder groups.

Of interest, clinical detection of antimicrobial resistant (AMR) pathogens colonizing wounds was found to be statistically significant between the Responder and Non-Responder groups, with higher levels of AMR pathogens found in Non-Responders, whether the patient had diabetes, prediabetes, or was non-diabetic. Other markers of overall health found to be statistically significant between Responder and Non-Responder groups include neuropathy, presence of peripheral artery disease (PAD), and osteomyelitis. Comorbidities associated with Responder versus Non-Responder were most notable in the patients with diabetes and included rates of amputation, smoking, hypertension, anemia, and hyperlipidemia.

### Single Block Model of Clinical Metrics, Microbiome, and Metabolome Features

A random selection of donors evenly distributed between Responders and Non-Responders was chosen for further metabolomics and microbiome profiling (Table 1). The major contributing features with statistical significance for each dataset are presented (Supplemental Figure 1). For the single block analysis of metabolite biomarkers, there were nineteen metabolites with statistically significant correlation to the Responder cohort. The xanthine alkaloid caffeine and its xanthosine derivative theophylline were the most highly correlated, statistically significant metabolites associated with wound response to care, i.e. the Responder cohort. Other significant metabolites of interest included several metabolites associated with fatty acid metabolism and vitamin B6 metabolism. On the Non-Responder metabolites, there are twenty-six statistically significant metabolites. The most significant is stachydrine, a proline derivative metabolite that has a role in host defense (29,30). Other significant metabolites (aminoadipic acid, D-ribose, histamine,1-methylhistamine, 3-methylhistidine, and D-chiro-inositol) have been associated with metabolic dysregulation and Type 2 diabetes (31–36). Finally, several lipids have significant statistical significance within the Non-Responder cohort (Supplemental Figure 1a). The microbiome data set includes fewer statistically significant features in the Responder and Non-Responder cohorts, with five bacterial predictors of a positive response to wound therapy and ten bacterial predictors of a poor response (Supplemental Figure 1b). The most statistically significant microbiome predictor for the Responder cohort is *Methylbacterium* and the most statistically significant microbiome predictor for the Non-Responder cohort is *Enterococcus*. For clinical variables, levels of triglycerides, total protein, and percent monocyte in peripheral blood were statistically significant features associated with a positive outcome for wound treatment, while fasting glucose, HemA1c, ARP, and hypertension had a statistically significant features were associated with the Non-Responder group, i.e. wounds recalcitrant to therapy (Supplemental Figure 1c).

### Integrative Model

Clinical care outcome was modeled as a function of metabolome, microbiome, and clinical datasets using DIABLO (**D**ata **I**ntegration **A**nalysis for **B**iomarker Discovery using **L**atent C**o**mponents) (1–3). DIABLO is a supervised algorithm based on partial least squares (PLS) that learns a multiomic predictive signature by maximizing the sum of covariances across datasets. DIABLO has a number of properties that make it well-suited to the present study. First, it limits overfitting and multicollinearity by projecting high-dimensional datasets into a much smaller set of *components*—uncorrelated linear combinations of the original features. Second, it performs LASSO feature selection (4), which allows for the identification of a subset of variables that are most important for predicting the outcome. Finally, DIABLO provides an integrated, systems-oriented solution by indicating how features from different datasets are correlated with each other—for example, that the presence of certain metabolites in a wound is highly predictive of the abundance of specific bacteria, and that these features together predict whether a wound will respond to standard of care.

Predictive performance in the final model resulted in a cross-validated error rate of 6.44%. Supplemental Table 3 and Supplemental Table 4 summarize the number of features selected by the model and integrated model error distribution. Out of 1,055 features modeled (metabolome, microbiome, and clinical markers), only 176 were required to label samples as either healing or non-healing with high accuracy. While error rate for all three single dataset models was within acceptable limits, the clinical markers dataset had the lowest error. The selected features in the model are provided in Supplemental Table 5.

**Figure 2:**
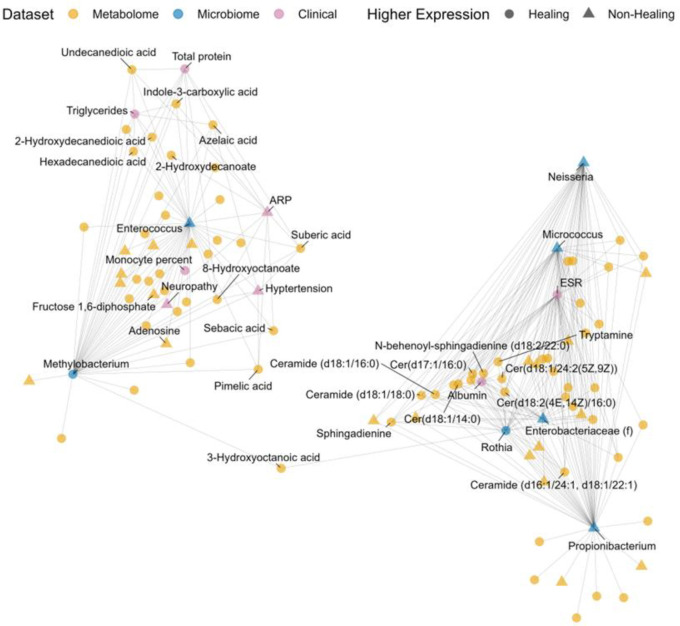
A network map representing the two-part multi-omic signature separated along Component 1 discovered by the model. Each point in the graph represents either a metabolome (orange), microbiome (blue), or clinical (purple) feature. Features with higher expression in healing wounds are plotted as circles; those with higher expression in non-healing wounds are plotted as triangles. Only model features with at least one correlation to another feature above/below ±0.45 are shown.

The multiomic signature can be displayed as a network-based model (Figure 2). The network representation displays only features that have correlations with at least one other feature above/below ±0.45. The two parts of the signature appear as separate groups, with Enterococcus, Methylobacterium and associated features on the left, and the ceramides, sphingadienines, Propionibacterium, Nesseria, and Micrococcus on the right. The network map clearly delineates model separation of features by correlation to Component 1 and Component 2, indicating limited connection between groupings within the threshold of between feature correlations above/below ±0.45.

**Figure 3:**
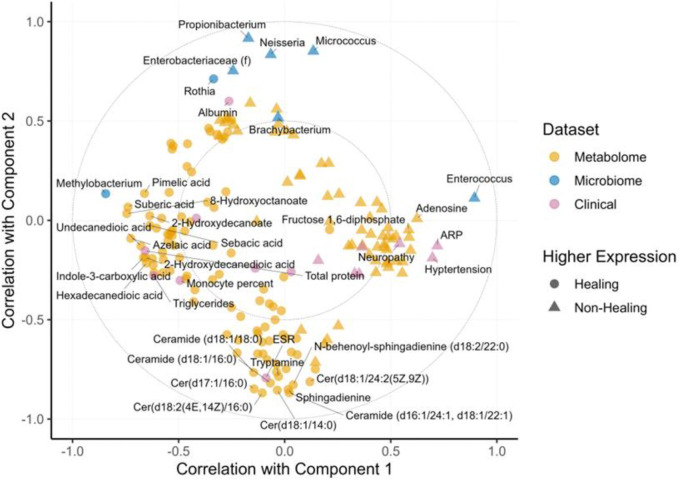
Circle plot illustrating the predictive two-part multi-omic signature discovered by the model. Four clear clusters of related features can be identified: Cluster 1 (C1) is centered around Methylobacterium; Cluster 2 (C2) is centered around Enterococcus; Cluster 3 (C3) centers around a series of metabolite features; and Cluster 4 (C4) centers around a group of microbial features. All 176 features selected into the model are located in 2D space according to their correlations with model components one (x-axis) and two (y-axis). The large dashed circles are guides representing correlations of ±0.5 (inner circle) and ±1 (outer circle). Features closer to the outer circle are more important for predicting the outcome. The angle made by connecting two features through the origin gives the sign of their correlation. Acute angles indicate positive correlations, obtuse angles represent negative correlations, and right angles indicate no correlation. Moreover, the length of the connecting lines gives the magnitude of the correlation.

The multiblock, integrative model can also be represented in circle plots (Figure 3). Circle plots represent features selected into the model, as well as relationships among features. Supplemental Figure 2 shows an example circle plot. Gray dots are model features plotted in 2D space, indicating their correlation with model components one (x-axis) and two (y-axis). The dashed circles are guides representing correlations of ±0.5 (inner circle) and ±1 (outer circle). Features closer to the outer circle are more important for predicting the outcome. The angle made by connecting two features through the origin gives the sign of their correlation. Acute angles indicate positive correlations (red and green), obtuse angles represent negative correlations (purple), and right angles indicate no correlation (blue). Moreover, the length of the connecting lines gives the magnitude of the correlation. For example, the angle made by the red and green lines is identical, but the red lines are longer, indicating a stronger positive correlation between the red features relative to the green features.

While the network map clearly indicates the separation of features between the two components of the model, the relationship between features is encoded in the circle plot, including which features are related to which features and the magnitude of that relationship. A circle plot with all 176 features selected into the model indicates a two-part multiomic signature (Figure 3). Four clear clusters of related features can be identified: Cluster 1 (C1) is centered around Methylobacterium and is oriented as negatively correlated to Component 1; Cluster 2 (C2) is centered around Enterococcus and is oriented as positively correlated with Component 1; Cluster 3 (C3) centers around a series of metabolite features and is oriented as negatively correlated with Component 2; and Cluster 4 (C4) centers around a group of microbial features and is oriented as positively correlated with Component 2. The first part of the multiomic signature is arrayed along the *x*-axis, Component 1, and is characterized by C1 and C2 positively correlated features on the right and left, which are associated with non-healing and healing wounds, respectively. Non-healing wounds have more Enterococcus, higher expression of metabolites like adenosine and fructose 1,6-diphosphate, and higher levels on clinical markers like ARP (antibiotic-resistant pathogens), hypertension, and neuropathy. In contrast, healing wounds have more Methylobacterium, higher expression of 8-hydroxyoctanoate, suberic acid, and azelaic acid, and higher total protein and triglyceride measurements. Importantly, features in the right and left clusters are positively correlated with their neighbors but negatively correlated with features in the opposite cluster. For example, higher abundance of Methylobacterium in a wound predicts lower adenosine levels, and higher abundance of Enterococcus predicts lower clinical metrics of total protein levels.

The second part of the signature is arrayed along the *y*-axis, Component 2, and is characterized by C3 and C4 of positively and negatively correlated features at the top and bottom of the figure, respectively. C3, at the bottom of the y-axis, contains ceramides, sphingadienines, tryptamine, and ESR (the erythrocyte sedimentation rate), all of which have higher expression in healing wounds. C4, at the top of the y-axis, is composed mostly of bacteria that are more abundant in non-healing wounds: Propionibacterium, Nesseria, and Micrococcus.

**Figure 4:**
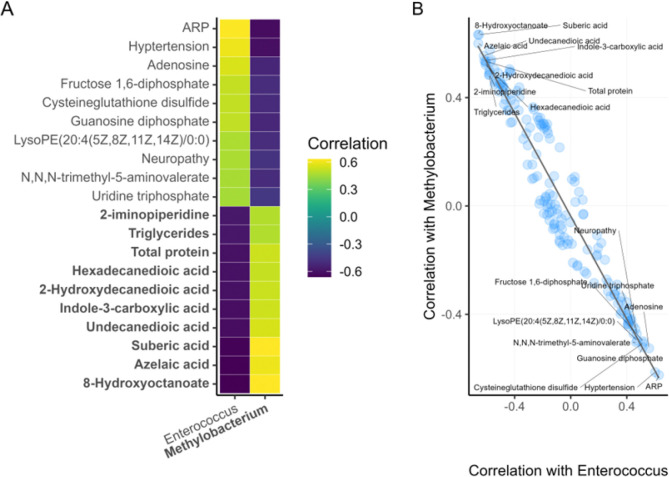
Enterococcus and Methylobacterium have mirror-image relationships to selected model features in the metabolome and clinical blocks. (A) shows the 20 metabolome and clinical features most strongly correlated with Enterococcus; correlations of the same features with Methylobacterium have similar magnitudes but flipped trends. Feature names written in bold have higher expression in healing wounds. (B) plots the correlations of all selected metabolite and clinical features (blue circles) with Enterococcus (x-axis) and Methylobacterium (y-axis). The black line indicates a linear fit with a slope of −0.95. All features shown on the y-axis in (A) are labeled in (B).

To further investigate the two part, multiomic signature of the integrative model, the metabolome and clinical features with positive and negative correlations to Enterococcus were plotted (Figure 4). The twenty features of highest positive and negative correlations were plotted on a heatmap and demonstrate that Methylobacterium has a striking mirror image relationship to the same features (Figure 4a). Plotting correlations with Methylobacterium on one axis and Enterococcus on the other axis establishes that this pattern persists across all 168 metabolome and clinical features. Remarkably, the linear fit shows that Methylobacterium correlations to other features can be accurately predicted by multiplying the Enterococcus correlations by −0.95 (Figure 4b).

**Figure 5:**
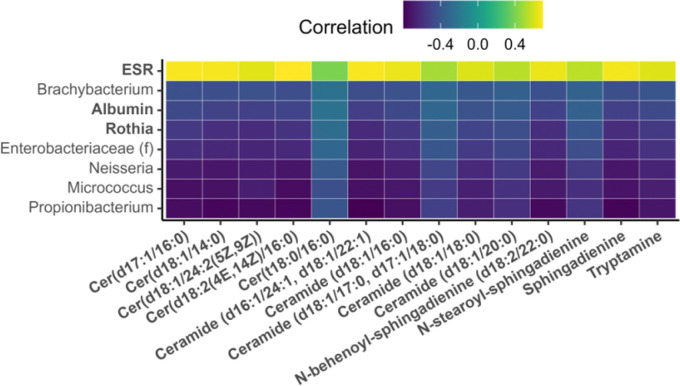
Heat map representing correlations between ceramides, sphingadienines, and tryptamine with microbiome and clinical features. Features are included on the y-axis of they have at least one correlation with an x-axis feature above/below ±0.35. Feature names printed in bold have higher expression in healing wounds. Parenthetical information after microbiome features indicates the taxonomic level at which feature identity was resolved—e.g., (f)amily, (o)rder. Microbiome features with no parenthetical information were resolved at the genus level.

As visualized in the network-based model representation (Figure 2), a second part of the multiomic signature clusters around ceramides, sphingadienines, and tryptamine. Plotting these correlations in a heatmap indicates very similar patterns of correlation to other model features. Specifically, ceramides, sphingadienines, and tryptamine are all positively correlated with ESR and negatively correlated with albumin and a set of microbiome features (with the exception of Rothia) that are more abundant in non-healing wounds (Figure 5).

## Metabolome Predictive Variables

**Figure 6:**
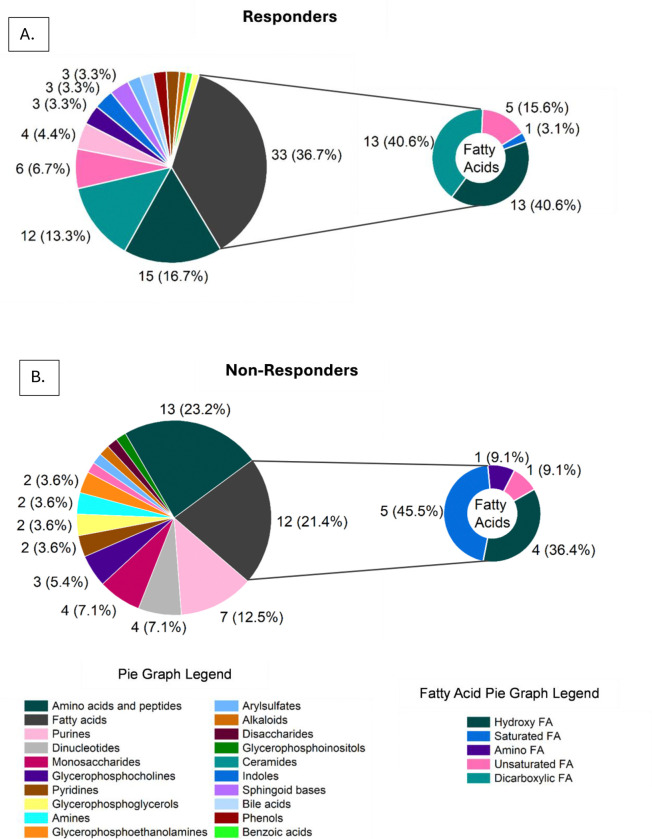
Metabolite features selected by sPLS-DA. Multiblock integrated model selected 153 metabolite features that associate with (A) Responder and (B) Non-Responder clinical outcome. Categorized metabolite features are shown with number of features per category and percent of category contribution to overall feature selection.

The sPLS-DA of the multiblock, integrated model identifies predictive variables that are both cross-correlated with clinical outcome (Responder versus Non-Responder) and provide insight into the metabolic microenvironment of the chronic wound. Derived from the multiblock model, 153 metabolite features were found to have predictive value for clinical outcome. The selected metabolite features were then categorized by metabolite type, with the number of metabolites selected per type and the percent contribution of that type to the overall metabolite features associated with clinical outcome shown (Figure 6). For both clinical outcomes, fatty acids were the most highly abundant metabolites selected and are highlighted separately. For the Responder clinical outcome, the highest contributing features to the model besides fatty acids were amino acids and peptides and ceramides at 16.7% and 13.3%, respectively (Figure 6A). For the Non-Responder clinical outcome, the highest contributing features to the model besides fatty acids were amino acids and peptides and purines at 21.4% and 12.5%, respectively (Figure 6B).

While fatty acid metabolites were a high percentage for both the Responder and Non-Responder outcomes at 36.7% and 21.4%, respectively, there were notable differences in the subclass of fatty acid metabolites (small pie graphs, Figure 6). For example, dicarboxylic fatty acids accounted for 40.6% of the fatty acids selected to predict healing, while no dicarboxylic fatty acids were selected for the Non-Responder clinical outcome. Furthermore, saturated fatty acids accounted for 45.5% of metabolites selected for the Non-Responder clinical outcome, but only 3.1% of this type of fatty acid was selected for the Responder clinical outcome. No amino fatty acids were selected for the Responder category, while 9.1% of the fatty acids selected for the Non-Responder category were amino fatty acids. A detailed list of metabolites can be found in Supplemental Table 6.

**Figure 7:**
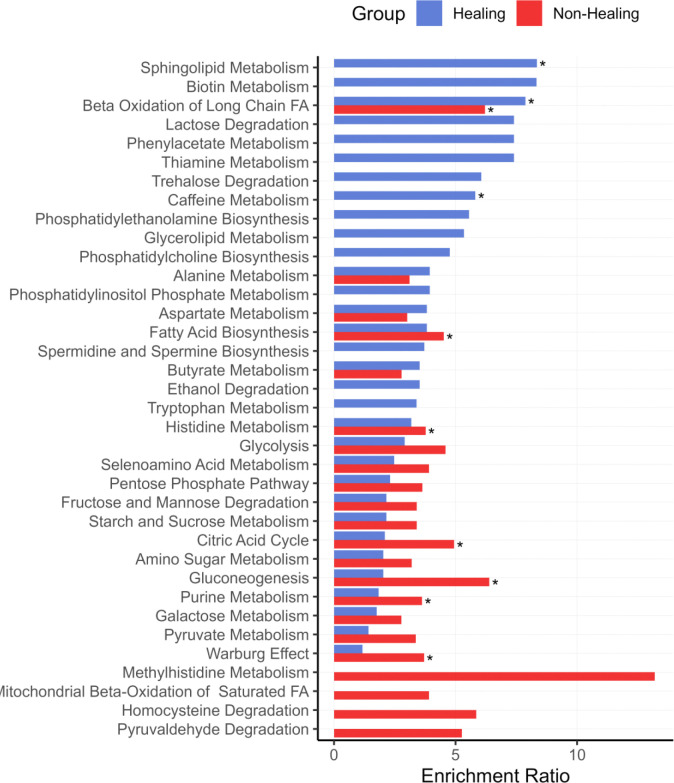
Metabolite set enrichment analysis (MSEA) of features selected by the sPLS-DA of the multiblock model. Metabolic pathways impacted by feature selection are shown for Healing (Blue) and Non-Healing (Red) wounds. The top 20 enriched pathways in each group are shown. Asterisks designate significantly enriched pathways.

The metabolic features selected by the model for each clinical outcome, Responder and Non-Responder, were then analyzed for metabolic pathway impact by metabolite set enrichment analysis (MSEA) to identify impacted pathways (Figure 7). For the Responder outcome, significantly impacted pathways included sphingolipid metabolism, beta oxidation of long chain fatty acids, and caffeine metabolism. For the Non-Responder outcome, significantly impacted pathways included beta oxidation of long chain fatty acids, fatty acid biosynthesis, histidine metabolism, citric acid cycle, gluconeogenesis, purine metabolism, and the Warburg effect. A detailed list of model selected metabolites and enrichment values can be found in Supplementary Tables 6 and 7.

## Microbiome Predictive Features

**Figure 8:**
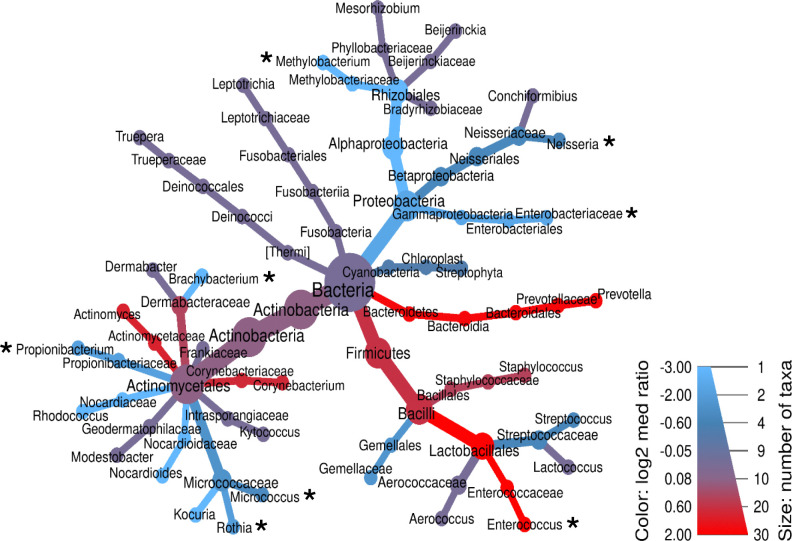
Heat tree showing relative abundance and phylogenetic relationships among 37 taxa with an absolute difference of 0.33 or higher, between Responders and Non-responders. The color indicates the relative numbers of taxa, those that are more abundant in non-responders (color more Red) and those more abundant in Responders (color more Blue). Microbial features selected by the integrated model are indicated by an asterisk (*).

As with metabolite features, the sPLS-DA identified predictive microbiome features associated with clinical outcome, healing versus non-healing wounds. There were 634 taxa identified in the full dataset and 180 microbial features were selected for model input. These taxa (identified down to genus, if possible, with the GG99 classifier) are shown in a heat tree diagram (Supplemental Figure 3), with relative abundance indicated by the color range. Branches and nodes colored toward blue are more abundant in Responders, while those colored redder are more abundant in the Non-Responder cohort. To more closely focus on microbiome features correlated with clinical outcome, a heat tree was constructed with 37 taxa that had a differential abundance of a factor of 0.3 or higher (Responder versus Non-Responder) after standardizing each microbial feature (Figure 8). In this focused heat tree, the eight microbial features selected by the model are indicated by asterisks. This heat tree highlights both the major players in the integrated model, as well as commonly cited microbes that are often regarded as major players contributing to non-healing but were not selected by the model as predictive of wound response to standard of care. Note that the two major microbial contributors to model separation along Component 1, Methylobacterium and Enterococcus, are clearly shown to have opposite abundance in healing versus non-healing wounds, respectively. Other microbial features selected by the model and found in Component 2 include Nesseria, Enterobacteriaceae, Brachybacterium, Propionibacterium, Micrococcus, and Rothia (Figure 8).

**Figure 9:**
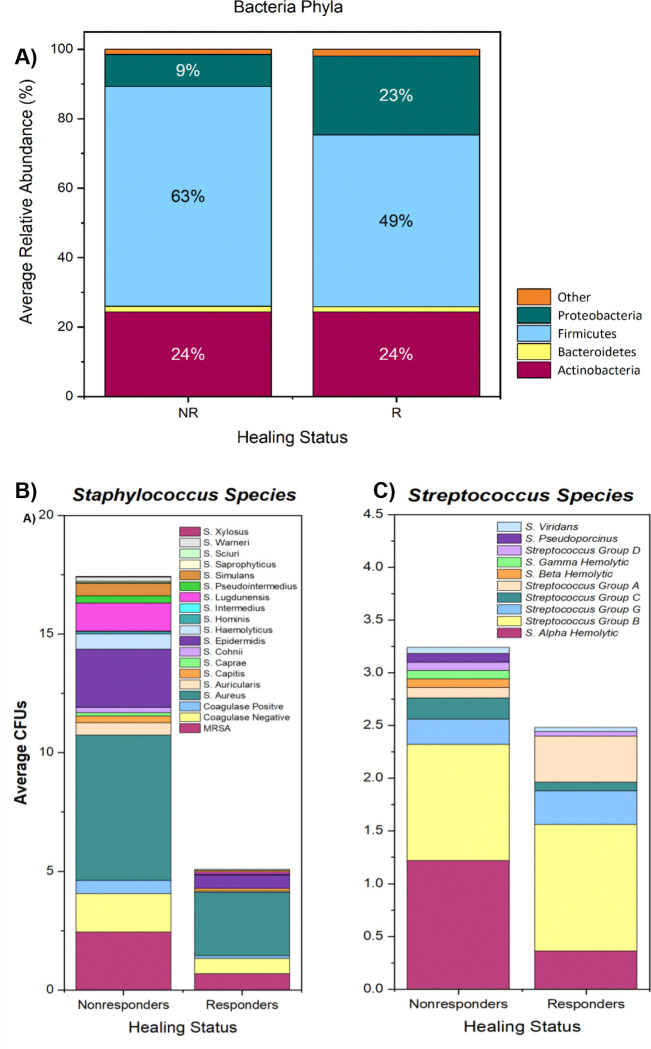
A) Stacked bar-chart showing relative abundance of major Phyla in Responders versus Nonresponders. Any phyla with mean abundances less than 1.5% were grouped into “other” category. B) Relative abundance of major Staphylococcus species and C) Streptococcus species identified by QIIME analysis and by BLAST’ing ASVs that were not identified to species level in QIIME workflow. Note that since the individual genera do not include all bacteria in the samples, the sum of mean relative abundance does not equal 100%. Species with mean abundances less than 1.5% were grouped into “Other”, along with OTUs not identified down to the species level.

Based on relative abundance (Figure 9), at the phylum level, the three main phyla present in both healing and non-healing wounds were Fimicutes, Actinobacteria, and Proteobacteria, which is consistent with previous wound healing studies. Bacteroidetes was the fourth most common phylum observed, having >1.5% abundance across all samples (Figure 9A). Phylum Firmicutes had greater abundance in the Non-Responder cohort and a large portion of this increase was due to the genera *Staphylococcus* and *Streptococcus.* In *Staphylococcus*, there was a greater abundance of *S. aureus* compared to other species in the Non-Responders; however, *S. aureus* was about twice as abundant as *S. epidermidis* in Responders (Figure 11B). In *Streptococcus*, a large proportion of the ASVs aligned with the *S. mitis* group and overrepresented in the Non-Responders. Among the *mitis* group taxa, a greater number were tentatively identified as *S. pneumoniae* in the Non-Responders. In addition, a greater number of taxa in the *S. salivarius* group were observed in the Non-Responder cohort (Figure 11C). Relative abundance in the Responder cohort had ~6% of *Streptococcus* identified as *S. dysgalactiae*, while none were found in the Non-Responders. The other phyla detected at a minimum of at least 0.03% of reads across the samples were: Acidobacteria, Abditibacteriota (previously FBP candidatus), Chloroflexi, Cyanobacteria, Deinococcota (Deinococcus-Thermus), Fusobacteria, Planctomycetes, and Verrucomicrobia. Interestingly, Bacterial community diversity (Faith’s PD) and evenness (Pilou’s evenness) were elevated in the Non-Responder cohort (data not shown).

## DISCUSSION

The primary objective of the present study was to inform understanding of wound healing in the study population by building a model that integrates across metabolome, microbiome, and clinical datasets. Our resulting DIABLO model has excellent predictive performance, correctly classifying 93.56% of held-out samples when evaluated using cross-validation. At the heart of the model is information about how the set of 176 selected features correlate not only with the outcome, but with one another. This knowledge is the key to integration and allows essential constraint of the space of hypotheses that can reasonably explain—at the systems level—why some wounds heal while others do not.

The model was oriented across two separate components designated as Component 1 and Component 2. Component 1, arrayed along the x-axis of the circle plot (Figure 3) contained two distinct clusters, cluster one (C1; Figure 3, left side) associated primarily with Responders and cluster two (C2; Figure 3, right side) corresponding with Non-Responders and highlighted the inverse relationship of *Methylobacterium* and *Enterococcus*, respectively. Arrayed along the y-axis, Component 2 encompassed the Responder associated cluster three (C3) and the Non-Responder centered cluster four (C4) which demonstrated the importance of ceramides within C3 (Figure 3, bottom) contrasted with the remaining microbiome features of the model in C4 (Figure 3, top).

The inverse distributions of *Methylobacterium* (C1) and *Enterococcus* (C2) are interesting findings of this study, worthy of follow-up in future studies. *Methylobacterium* is commonly encountered in human gut microbiome studies (37) and is a common soil and rhizosphere (plant root associated) bacterium, and has been found in relatively large quantities in other vertebrate gut microbiomes (38). Despite its general classification as an environmental commensal bacterium (39), *Methylobacterium* presence in Responder wounds may suggest a benign microbiome within healing wounds (40,41). Although *Methylobacterium* has been suspected of being a contaminant in some microbiome studies (42), its non-random distribution in our samples, showing up in appreciable amounts almost exclusively in Responders, points to it being an actual constituent of the microbiome of these wounds. Metabolically, C1 contained an abundance of medium chain and long chain dicarboxylic acids (DAs) (i.e. pimelic, suberic, azelaic, sebacic, dodecanedioic, undecanedioic, tetradecanedioic, hexadecanedioic, plus 8-hydroxyoctanoate) and methylxanthine and alkaloid metabolites (i.e. caffeine, theophylline, trigonelline, and vanillin). DAs are a biproduct of active ω-oxidation followed by peroxisomal β-oxidation which facilitates lipid cleanup and fatty acid oxidation when cells are under lipid metabolic stress (43). The resulting DAs, especially azelaic acid, exert antimicrobial and anti-inflammatory actions in skin (43–45). The co-occurrence of the total protein and monocyte percent clinical markers within this healing cluster suggests a better nourished, immune-competent state where macrophages can orchestrate the transition from inflammation to repair, and higher triglycerides likely reflect available energy substrates needed for remodeling rather than the maladaptive dyslipidemia seen in non-healers (46–49). A second, highly distinctive element in C1 is the methylxanthine and alkaloid signature which may contribute to a healing environment via systemic benefits as habitual caffeine intake has been linked to better insulin sensitivity and lower inflammationb(50,51) and through local signaling pathways which temper inflammationb(52–54).

*Enterococcus* species, the dominant feature in the Non-Responder associated C2, are known prolific biofilm formers in chronic wounds that contribute to slow healing, resist antibiotic treatment, modulate host immunity, and sustain a persistent low inflammatory state (55,56). Mechanistically, this is supported by metabolomic and clinical features that indicate a wound environment driven by hyperglycemic-fueled metabolic stress and non-healing. The clinical marker profile, characterized by elevated ESR, high HbA1c, neuropathy, hypertension, and antibiotic-resistant organisms, reflects the classic high-risk DFU phenotype (57–59). This assertion was also evidenced by the presence of D-ribose (31), 2-aminoadipic acid (32), and 5-(galactosylhydroxy)-L-lysine (60) all of which are indicative of diabetic risk. The metabolites 2-methylbutyroylcarnitine and N,N,N-trimethyl-5-aminovalerate, which originates from microbial trimethyllysine, point towards the inhibition of carnitine biosynthesis and incomplete fatty-acid oxidation (61–64). Additionally, the presence of cysteine-glutathione disulfide which flags oxidative stress, and 3-methylhistidine and N-acetyl-3-methylhistidine which reflect muscle/ECM proteolysis further supports a non-healing phenotype (65,66). Finally, the presence of chronically elevated histamine, associated with vasodilation, increased vascular permeability, and localized inflammation, might indicate the presence of ineffective inflammation and persistent infection (67,68).

Within Component 2, ceramides, ceramide precursors, and sphingoid bases, represented 31% of the metabolic features selected for C3. Together, these metabolites are known to drive sphingolipid and ceramide metabolism which supply critical components needed for keratinocyte migration and differentiation during the proliferative and remodeling phases of wound healing (69–71). Additionally, tryptamine, a tryptophan metabolite in both microbial and host metabolism, has been shown to activate signaling receptors in keratinocytes and reduce inflammation in skin disorders to facilitate healing (72,73). The co-occurrence of ESR, elevated in healers, is noteworthy as a systemic inflammation marker within this cluster. This trend which has been reported in other research (74) suggests that patients with healing wounds have a higher systemic inflammatory response which is beneficial within the healing process. Alternatively, the lower ESR levels in Non-Responders might suggest an ineffective systemic response that results in a smoldering localized inflammation that never fully engages the healing process (75).

In contrast, C4 epitomized a dysbiotic wound ecology governed by biofilm forming facultative skin colonizers including *Cutibacterium, Micrococcus, Neisseria, Enterobacteriaceae*, which have the capacity to become pathogenic depending on the species present and host immunity (76–79). Fifty five percent of the C4 metabolites are medium chain fatty acids (MCFAs) including heptanoic, pelargonic, 3-Hydroxyhexanoic, caprylic, and caproic acid. The MCFAs heptanoic, 3-Hydroxyhexanoic, and pelargonic acid along with allantoic acid are associated with microbial metabolism and have been identified as potential biomarkers for impaired glucose regulation and diabetes (80,80–82). Additionally, many of these MCFAs have been associated with systemic or localized inflammation (83–86). Two features of this cluster were higher in Responders, the clinical feature of albumin and the microbe *Rothia.* Albumin, a robust marker of nutrition, normally coincides with higher total protein levels that also correlated with the Responder cohort (87). Conversely, low albumin has been shown to correlate with delayed wound closure following surgical wound debridement or amputation (34). *Rothia*, a common microbe in the oral mucosa, is a high-capacity nitrate reducer that has been linked to increased healing in periodontitis and chronic lung disease (88,89); however, its role in chronic wounds is largely unknown.

Viewed through the lens of chronic wounds in T2DM, this data resolved into two largely orthogonal components. Along Component 1, C1 at one end defined a metabolically competent wound containing benign microbial colonizers within a nutritionally resourced and immunologically capable host. Conversely, C2 embodied the classic high-risk DFU patient with non-healing wounds infected with pathogenic biofilms and with the metabolic signature of a wound stuck in breakdown mode. Translationally, the model suggests that moving a non-healing patient from C2 to C1 would involve tight glycemic control, nutritional optimization and support, and aggressive infection/biofilm management. Within Component 2, C3 represented a reparative wound dominated by barrier lipid remodeling and effective systemic inflammation balanced by anti-inflammatory signatures. In contrast, C4 alluded to the presence of polymicrobial biofilms and microbial metabolites that contribute to sustained inflammation. Therefore, to move a patient in C4 towards the reparative C3 might involve treatment to decrease polymicrobial infections, intervention to increase barrier lipid remodeling along with nutritional support. Importantly, the two independent, orthogonal components intimate that a patient can be well controlled metabolically (towards C1) yet can still stall locally (towards C4). Furthermore, the mechanistic story that emerges is clinically intuitive, suggesting that healing happens when host metabolism, nutrition, and immunity align with a locally pro-repair lipid and microbiome environment. Conversely, chronic wounds persist when pathogenic biofilms and microbial-dominated wound biochemistry overpowers a nutritionally depleted, hyperglycemic host.

Through this work we have demonstrated that the complex wound microenvironment coupled with the metabolic health of the patient can be combined within an integrated computer model to predict wound healing. There are, however, limitations to this study. First, this study is constrained by the limitations of our cohort within our local Veteran population which is predominately Caucasian and male. Additional studies will be needed to ensure that these findings correlate to a larger ethnically diverse and gender-balanced cohort. Furthermore, this model was created from a smaller sample size and the addition of more samples will be needed to further refine the model. Finally, this integrative model is hypothesis generating, giving deep insight into the cellular and molecular contributions to non-healing; however, these insights are correlative and further mechanistic studies to establish causation are needed.

## Supplementary Material

Supplement 1

## Figures and Tables

**Figure 1:**
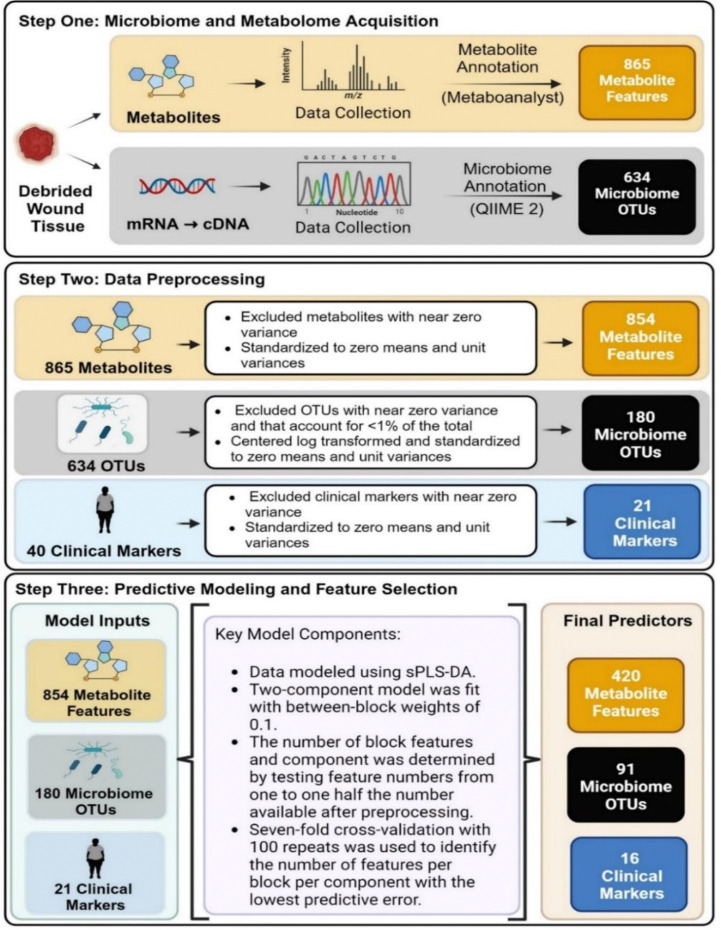
Analytical overview of the predictive model using the metabolome, microbiome, and clinical marker feature sets. First, localized chronic wound metabolite profile and colonizing microbiome OTUs were collected and annotated utilizing Metaboanalyst 5.0 and QIIME 2. Second, data preprocessing steps were applied to eliminate near zero variance and to standardize the metabolome, microbiome, and clinical marker feature sets. Third, the predictive model was applied to select features that could predict healing status with the lowest error rates.

**Table 1:** Demographic and Clinical Characterization of patient cohort for which debridement tissue samples were characterized for microbiome and metabolome.

Total Samples (N = 45)	Responders (n = 20) Mean ± SE or n (%)	Non-Responders (n = 25) Mean ± SE or n (%)

Avg Age (years)	70.7 ± 3.1	70.4 ± 1.6
BMI (kg/m^2^)	34.6 ± 1.8	31.1 ± 0.9
Glucose (mg/dL)*	152 ± 8.9	194 ± 9.2
Wound Size (cm^3^)	4.4 ± 1.6	1.2 ± 0.2
Wound Depth (cm)*	0.5 ± 0.1	0.2 ± 0.02
Avg AMR Pathogens*	1.7 ± 0.4	3.8 ± 0.5
HA1C*	6.1 ± 0.3	7.0 ± 0.3
HO Ampuation*	0 (0)	10 (40)
HO Smoking	11 (55)	13 (52)
HO Hypertension*	12 (60)	24 (96)
HO Anemia	5 (25)	8 (32)
HO Hyperlipedemia	13 (65)	19 (76)
HO Neuropathy*	4 (20)	17 (68)
HO PAD*	1 (5)	6 (24)
***HO Osteomyelitis*** *	1 (5)	10 (40)
Albumin	3.7 ± 0.1	3.7 ± 0.1
Alkaline Phosphotase	83.3 ± 4.6	91.7 ± 2.5
CRP	2.4 ± 0.8	1.1 ± 0.3
ESR	33.7 ± 4.2	24.1 ± 3.2
Monocyte %*	11.3 ± 0.7	7.7 ± 0.5
Total Protein*	7.4 ± 0.1	6.9 ± 0.1
Triglycerides*	180 ± 15	90 ± 8.0

Data was extracted from the patient electronic medical record. Clinical markers determined significantly different between Responders and Non-Responders by are designated on the chart in bold.
