# Supplementary material for: Multiblock Integration and Modeling of Localized Microbiome, Metabolome, and Clinical Metadata to Identify Biomarkers Predictive of Outcome in Veterans with Non-Healing Wounds
Source: bioRxiv. 2025 Sep 10:2025.09.07.674782. Preprint. [Version 1] doi: 10.1101/2025.09.07.674782 (PMC12439902; doi:10.1101/2025.09.07.674782)
Supplement: Supplementary file 1 [file media-1.pdf]

SUPPLEMENTAL FIGURES/TABLES

Supplemental Table 1:

**Total Participants**	**Responders (n = 25)** Mean ± SE or n (%)			**Non-Responders (n = 50)** Mean ± SE or n (%)		
n = 75	Diabetic 10 (40.0%)	Prediabetic 10 (40.0%)	Nondiabetic 5 (20%)	Diabetic 40 (80%)	Prediabetic 6 (12%)	Nondiabetic 4 (8%)
Avg Age (years)	70.7 ± 3.1	72.6 ± 2.9	73.6 ± 5.7	70.4 ± 1.6	73.7 ± 1.4	79 ± 3.1
BMI (kg/m^2)	34.6 ± 1.8	26.8 ± 1.6	26.7 ± 3.2	31.1 ± 0.9	31.1 ± 2.1	27.9 ± 3.7
Glucose (mg/dL)*	152 ± 8.9	111 ± 4.1	101.2 ± 4.7	194 ± 9.2	111 ± 3.7	114 ± 3.8
Wound Size (cm^3)	27.4 ± 13.1	28.6 ± 17.4	1.99 ± 0.9	19.1 ± 6.3	20.6 ± 11.1	13.5 ± 10.6
Wound Depth (cm)	1.2 ± 0.5	1.0 ± 0.3	0.6 ± 0.2	1.1 ± 0.2	1.1 ± 0.4	1.2 ± 0.7
Avg AMR Pathogens*	1.9 ± 0.4	1.2 ± 0.6	1.00 ± 0.6	4.7 ± 0.4	3.3 ± 1.2	3.3 ± 0.8
HA1C	7.2 ± 0.4	5.9 ± 0.9	5.12 ± 0.1	8.5 ± 0.3	6.1 ± 0.1	5.4 ± 0.1
HO Ampuation*	0 (0)	0 (0)	0 (0)	29 (72.5)	3 (50)	1 (25)
HO Smoking	2 (20)	6 (60)	4 (80)	17 (42.5)	2 (33.3)	2 (50)
HO Hypertension	8 (80)	4 (40)	4 (80)	32 (80)	3 (50)	4 (100)
HO Anemia	2 (20)	3 (30)	2 (40)	9 (22.5)	2 (33.3)	2 (50)
HO Hyperlipedemia	7 (70)	7 (70)	2 (40)	35 (87.5)	3 (50)	1 (25)
HO Neuropathy*	6 (60)	5 (50)	2 (40)	36 (90)	6 (100)	4 (100)
HO PAD*	1 (10)	1 (10)	2 (40)	23 (57.5)	5 (83.3)	3 (75)
HO Osteomyelitis*	1 (10)	2 (20)	0 (0)	28 (70)	2 (33.3)	1 (25)

Supplement Table 3: Number of features selected and errors for the best-performing single dataset sPLS-DA models.

Dataset	Measured	Modeled	Component 1 Selected	Component 2 Selected	Unique Selected
Metabolome	865	854	80	80	153
Microbiome	634	180	2	6	8
Clinical	21	21	10	10	15
Total	1,520	1,055	92	96	176

[illegible]pplement Table 4: Integrated multiblock err[illegible] [illegible]ased on sevenfold cross-validation with 100 repeats.

dist	comp1	comp2
max.dist	0.104	0.065
centroids.dist	0.107	0.083
mahalanobis.dist	0.107	0.064

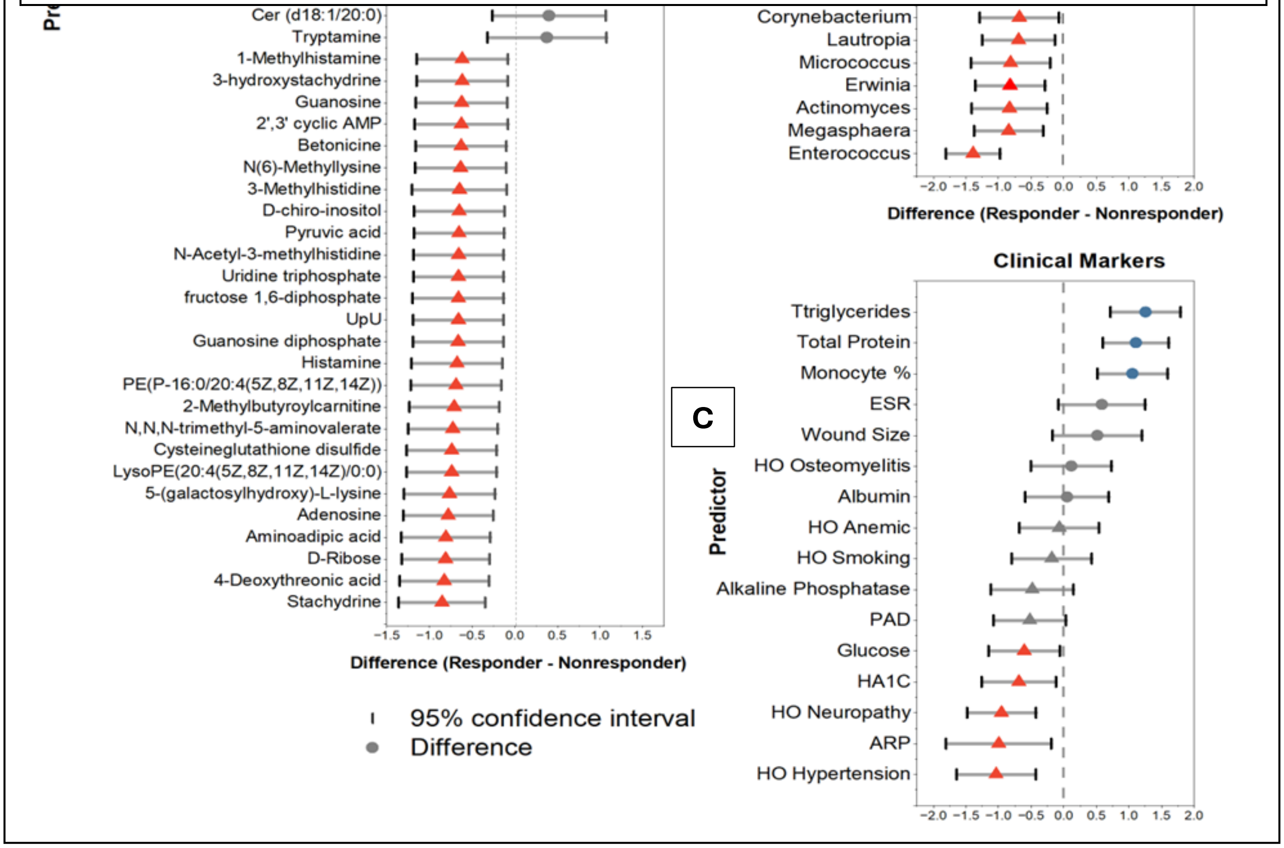

Supplemental Table 5: Selected model features ordered by dataset, component, and absolute value of the loading weight.

Dataset	Feature	Cluster	Comp1 Weight	Comp2 Weight	Comp 1 Cor	Comp 2 Cor
Microbiome	Methylobacterium	1	-0.6403	0.0000	-0.84	0.13
Metabolome	8-Hydroxyoctanoate	1	-0.1224	0.0000	-0.74	0.03
Metabolome	Suberic acid	1	-0.1758	0.0000	-0.74	0.07
Metabolome	Azelaic acid	1	-0.1426	0.0000	-0.73	-0.09
Metabolome	Undecanedioic acid	1	-0.1722	0.0000	-0.68	-0.13
Metabolome	Indole-3-carboxylic acid	1	-0.0467	0.0000	-0.67	-0.19
Metabolome	Pimelic acid	1	-0.1777	0.0000	-0.66	0.14
Clinical	Total protein	1	-0.4365	-0.0872	-0.66	-0.15
Metabolome	2-Hydroxydecanedioic acid	1	-0.0482	0.0000	-0.66	-0.19
Metabolome	Hexadecanedioic acid	1	-0.1070	0.0000	-0.65	-0.21
Metabolome	2-Hydroxydecanoate	1	-0.0372	0.0000	-0.64	0.02
Metabolome	Sebacic acid	1	-0.1237	0.0000	-0.64	-0.03
Metabolome	Formylanthranilic acid	1	-0.0328	0.0000	-0.62	-0.19
Metabolome	Valyl-Glutamine	1	-0.0668	0.0000	-0.62	-0.23
Clinical	Triglycerides	1	-0.3511	0.0000	-0.62	-0.28
Metabolome	2-iminopiperidine	1	-0.0347	0.0000	-0.62	-0.26
Metabolome	Tetradecanedioic acid	1	-0.1407	0.0000	-0.60	-0.04
Metabolome	Dodecanedioic acid	1	-0.1243	0.0000	-0.58	0.01
Metabolome	Bicine	1	-0.0412	0.0000	-0.58	-0.16
Metabolome	Methylguanidine	1	-0.0322	0.0000	-0.58	-0.26
Metabolome	Octadecenedioate (c18:1-dc)	1	-0.0402	0.0000	-0.57	-0.20
Metabolome	O-methoxycatechol-O-sulphate	1	-0.0084	0.0000	-0.55	-0.07
Metabolome	N-Acetyl-beta-alanine	1	-0.0979	0.0000	-0.55	-0.10
Metabolome	3-Hydroxyisovaleric acid	1	-0.0793	0.0000	-0.54	0.14
Metabolome	Dimethylmalonic acid	1	-0.1708	0.0000	-0.54	-0.07
Metabolome	Malonic acid	1	-0.0398	0.0000	-0.54	-0.25
Metabolome	Glutamine conjugate of c6h10o2 (2)	1	-0.0268	0.0000	-0.53	-0.06
Metabolome	Pregnenetriol sulfate	1	-0.0405	0.0000	-0.53	-0.08
Metabolome	3-Hydroxycapric acid	1	-0.0424	0.0000	-0.53	-0.05
Metabolome	1H-Indole-3-carboxaldehyde	1	-0.0085	0.0000	-0.52	-0.13
Metabolome	3-hydroxy-2-methylpyridine sulfate	1	-0.1264	0.0000	-0.49	-0.19
Clinical	Monocyte percent	1	-0.4248	-0.1993	-0.49	-0.30
Metabolome	Glutamine conjugate of c7h12o2	1	-0.0003	0.0000	-0.49	0.02
Metabolome	Cinnamoylglycine	1	-0.0513	0.0000	-0.47	-0.05
Metabolome	Acetylglycine	1	-0.0048	0.0000	-0.47	-0.29
Metabolome	Formiminoglutamic acid	1	-0.0225	0.0000	-0.46	-0.28
Metabolome	Vanillin	1	-0.1033	0.0318	-0.46	0.27
Metabolome	Hydroxyoctanoic acid	1	-0.0422	0.0326	-0.46	0.39
Metabolome	2-Furoylglycine	1	-0.0625	0.0000	-0.45	-0.01
Metabolome	Adipic acid	1	-0.0574	0.0000	-0.44	0.24
Metabolome	Caffeine	1	-0.2728	0.0000	-0.43	-0.41
Metabolome	N-Acetylasparagine	1	-0.0004	0.0000	-0.42	-0.35
Microbiome	Enterococcus	2	0.7681	0.0000	0.89	0.11
Clinical	ARP	2	0.2319	0.0000	0.72	-0.13
Clinical	Hyptertension	2	0.4915	-0.3627	0.70	-0.19
Metabolome	Adenosine	2	0.1593	0.0000	0.62	0.01
Metabolome	Guanosine diphosphate	2	0.1056	0.0000	0.59	-0.15
Metabolome	Cysteineglutathione disulfide	2	0.1665	0.0000	0.59	-0.09

Metabolome	Fructose 1,6-diphosphate	2	0.0834	0.0000	0.58	-0.05
Metabolome	LysoPE(20:4(5Z,8Z,11Z,14Z)/0:0)	2	0.1532	0.0000	0.56	-0.21
Metabolome	N,N,N-trimethyl-5-aminovalerate	2	0.1600	0.0000	0.55	-0.27
Clinical	Neuropathy	2	0.3251	0.0000	0.54	-0.12
Metabolome	PE(P-16:0/20:4(5Z,8Z,11Z,14Z))	2	0.0982	0.0000	0.53	-0.21
Metabolome	Uridine triphosphate	2	0.1064	0.0000	0.52	-0.08
Metabolome	2-Methylbutyroylcarnitine	2	0.1374	0.0000	0.51	-0.09
Metabolome	Stachydrine	2	0.2701	0.0000	0.49	-0.24
Metabolome	Guanosine triphosphate	2	0.0305	0.0000	0.49	-0.04
Metabolome	3-Methylhistidine	2	0.0850	0.0000	0.49	-0.08
Metabolome	N-Acetyl-3-methylhistidine	2	0.1167	0.0000	0.48	-0.21
Metabolome	3-hydroxystachydrine	2	0.0993	0.0000	0.48	-0.15
Metabolome	Fibrinopeptide B (1-12)	2	0.0320	0.0000	0.48	-0.22
Metabolome	2',3' cyclic AMP	2	0.0339	0.0000	0.47	-0.14
Metabolome	Guanosine	2	0.0396	0.0000	0.47	-0.12
Metabolome	D-chiro-inositol	2	0.1139	0.0000	0.46	-0.08
Metabolome	Aminoadipic acid	2	0.2104	0.0000	0.46	0.06
Metabolome	1-Methylhistamine	2	0.0359	0.0000	0.44	-0.13
Metabolome	D-Ribose	2	0.1873	0.0221	0.44	0.09
Metabolome	N(6)-Methyllysine	2	0.0723	0.0000	0.43	-0.26
Metabolome	Histamine	2	0.0945	0.0000	0.43	-0.20
Metabolome	Cer(d18:2(4E,14Z)/16:0)	3	0.0000	-0.2150	-0.11	-0.87
Metabolome	Ceramide (d16:1/24:1, d18:1/22:1)	3	0.0000	-0.2073	0.02	-0.87
Metabolome	Cer(d18:1/14:0)	3	0.0000	-0.1862	-0.03	-0.85
Metabolome	Sphingadienine	3	0.0000	-0.2078	0.02	-0.85
Metabolome	Cer(d17:1/16:0)	3	0.0000	-0.2195	-0.14	-0.85
Metabolome	N-behenoyl-sphingadienine (d18:2/22:0)	3	0.0000	-0.1907	0.04	-0.83
Metabolome	Ceramide (d18:1/16:0)	3	0.0000	-0.1405	-0.07	-0.82
Metabolome	Cer(d18:1/24:2(5Z,9Z))	3	0.0000	-0.1733	0.12	-0.81
Clinical	ESR	3	0.0000	-0.6713	-0.09	-0.79
Metabolome	Tryptamine	3	0.0000	-0.2220	-0.03	-0.79
Metabolome	Ceramide (d18:1/18:0)	3	0.0000	-0.1625	-0.15	-0.77
Metabolome	Hexadecasphingosine (d16:1)	3	0.0000	-0.1491	-0.04	-0.76
Metabolome	2-dehydro-3-deoxy-D-gluconic acid	3	0.0000	-0.2263	0.14	-0.75
Metabolome	2'-deoxyadenosine 3'-monophosphate	3	0.0000	-0.1640	0.01	-0.75
Metabolome	13Z,16Z-docosadienoic acid	3	0.0000	-0.1076	-0.04	-0.72
Metabolome	3-methyl pyruvic acid	3	0.0000	-0.1058	0.14	-0.71
Metabolome	Eicosenoic acid	3	0.0000	-0.0775	-0.08	-0.70
Metabolome	N(2)-Acetyllysine	3	0.0000	-0.1534	0.06	-0.68
Metabolome	Ceramide (d18:1/20:0)	3	0.0000	-0.1263	-0.10	-0.67
Metabolome	Nonadeca-10(Z)-enoic acid	3	0.0000	-0.0211	-0.13	-0.67
Metabolome	N-stearoyl-sphingadienine	3	0.0000	-0.1097	-0.22	-0.67
Metabolome	LysoPG(16:0/0:0)	3	0.0000	-0.0597	0.03	-0.66
Metabolome	N2-acetyl,N6,N6-dimethyllysine	3	0.0000	-0.0662	0.19	-0.62
Metabolome	N-Acetylglutamic acid	3	0.0000	-0.1289	-0.11	-0.61

Metabolome	Erucic acid	3	0.0000	-0.0818	-0.07	-0.61
Metabolome	Ceramide (d18:1/17:0, d17:1/18:0)	3	0.0000	-0.0767	-0.22	-0.60
Metabolome	LysoPG(18:1(9Z)/0:0)	3	0.0000	-0.1367	0.20	-0.60
Metabolome	Sphingosine	3	0.0000	-0.0440	0.06	-0.59
Metabolome	Piperine	3	0.0000	-0.1089	0.01	-0.59
Metabolome	PC(P-16:0/16:1(9Z))	3	0.0000	-0.0908	-0.01	-0.58
Metabolome	Phenylalanyl-Alanine	3	0.0000	-0.0036	-0.27	-0.58
Metabolome	10Z-Heptadecenoic acid	3	0.0000	-0.0052	-0.13	-0.57
Metabolome	Histidinyl-Alanine	3	0.0000	-0.0355	-0.13	-0.56
Metabolome	PC(P-16:0/16:0)	3	0.0000	-0.0059	0.08	-0.55
Metabolome	2-Hydroxy-3-methylbutyric acid	3	0.0000	-0.0740	-0.09	-0.54
Metabolome	LysoPG(18:2(9Z,12Z)/0:0)	3	0.0000	-0.0066	0.25	-0.53
Metabolome	Palmitoleic acid	3	0.0000	-0.0187	-0.05	-0.50
Metabolome	Cer(t18:0/16:0)	3	0.0000	-0.0029	-0.21	-0.48
Metabolome	2-hydroxynervonate	3	0.0000	-0.0413	-0.02	-0.46
Metabolome	MG(16:1(9Z)/0:0/0:0)	3	-0.0622	-0.1618	-0.25	-0.44
Metabolome	N-Acetylcitrulline	3	0.0000	-0.0771	-0.05	-0.43
Microbiome	Propionibacterium	4	0.0000	0.6370	-0.17	0.92
Microbiome	Micrococcus	4	0.0000	0.4608	0.13	0.85
Microbiome	Neisseria	4	0.0000	0.4939	-0.07	0.83
Microbiome	Enterobacteriaceae (f)	4	0.0000	0.3565	-0.24	0.75
Microbiome	Rothia	4	0.0000	0.0945	-0.34	0.71
Clinical	Albumin	4	0.0000	0.4036	-0.26	0.60
Metabolome	Heptanoic acid	4	0.0000	0.1898	-0.16	0.59
Metabolome	Caproic acid	4	0.0000	0.1524	-0.04	0.56
Microbiome	Brachybacterium	4	0.0000	0.0443	-0.03	0.52
Metabolome	DL-3-hydroxy caproic acid	4	0.0000	0.1835	-0.28	0.52
Metabolome	Alpha-Ketooctanoic acid	4	0.0000	0.1640	-0.27	0.51
Metabolome	(R)-Pantoic acid	4	0.0000	0.1131	-0.25	0.50
Metabolome	Pelargonic acid	4	0.0000	0.1914	-0.31	0.50
Metabolome	Caprylic acid	4	0.0000	0.1988	-0.28	0.49
Metabolome	Allantoic acid	4	0.0000	0.1306	-0.03	0.48
Metabolome	PC(18:2(9Z,12Z)/18:2(9Z,12Z))	4	0.0000	0.0918	-0.24	0.48
Metabolome	MG(16:0/0:0/0:0)	4	0.0000	0.0330	-0.36	0.46
Metabolome	MG(16:3)	4	0.0000	0.0035	-0.22	0.45
Metabolome	MOPS	4	0.0000	0.0659	0.01	0.45
Metabolome	MG(0:0/16:0/0:0)	4	0.0000	0.0211	-0.35	0.45
Metabolome	MG(0:0/15:0/0:0)	4	0.0000	0.0032	-0.27	0.45
Metabolome	Butyric acid	4	0.0000	0.0838	-0.05	0.44
Metabolome	MG(0:0/14:0/0:0)	4	0.0000	0.0113	-0.32	0.43
Metabolome	Thioproline	4	0.0000	0.1299	0.04	0.43
Metabolome	(2 or 3)-decenoate (10:1n7 or n8)	4	0.0000	0.0842	-0.31	0.42
Clinical	HA1C		0.2623	0.0000	0.37	-0.13
Metabolome	De(carboxymethoxy) cetirizine acetic acid		-0.2413	0.0000	-0.28	0.14
Metabolome	Theophylline		-0.2395	0.0000	-0.34	-0.30
Metabolome	4-Deoxythreonic acid		0.2218	0.0455	0.26	0.13
Metabolome	3-Hydroxypyridine sulfate		-0.1693	0.0000	-0.34	-0.13
Clinical	Glucose		0.1598	-0.0251	0.33	-0.27

Metabolome	5-(galactosylhydroxy)-L-lysine		0.1435	0.0000	0.40	0.01
Metabolome	Trigonelline		-0.1272	0.0000	-0.33	0.07
Clinical	PAD		0.1002	-0.3038	0.35	-0.27
Metabolome	Pyruvic acid		0.0888	0.0000	0.32	-0.12
Metabolome	Betonicine		0.0482	0.0000	0.40	-0.17
Metabolome	UpU		0.0449	0.0023	0.36	0.09
Metabolome	N1,N8-Diacetylspermidine		0.0411	0.0000	0.30	-0.13
Metabolome	GpA		0.0367	0.0000	0.41	-0.07
Metabolome	P-cresol glucuronide		-0.0335	0.0000	-0.36	0.09
Metabolome	4-Allylphenol sulfate		0.0287	0.0000	0.21	-0.01
Metabolome	Acesulfame		0.0276	0.0000	0.37	-0.13
Metabolome	ApG		0.0246	0.0000	0.36	-0.05
Clinical	Wound depth		-0.0171	0.0000	-0.42	0.01
Metabolome	Quinic acid		-0.0159	0.0000	-0.25	-0.16
Metabolome	ADP-ribose		0.0143	0.0201	0.21	0.29
Metabolome	Taurochenodesoxycholic acid		-0.0055	0.0000	-0.31	-0.27
Clinical	Osteomyelitis		0.0000	-0.2750	0.03	-0.26
Clinical	Wound size		0.0000	-0.1682	-0.14	-0.24
Metabolome	Capric acid		0.0000	0.1075	-0.53	0.36
Clinical	Anemia		0.0000	-0.1036	0.16	-0.20
Metabolome	3-Hydroxyoctanoic acid		0.0000	0.0865	-0.54	0.39
Metabolome	2-hydroxyheptanoate		0.0000	0.0828	-0.53	0.37
Metabolome	Sucrose		0.0000	0.0742	0.17	0.29
Metabolome	PC(18:2(9Z,12Z)/20:4(5Z,8Z,11Z,14Z))		0.0000	0.0730	-0.29	0.42
Metabolome	Methyl-4-hydroxybenzoate sulfate		0.0000	-0.0623	0.00	-0.28
Metabolome	Adenosine triphosphate		0.0000	-0.0439	0.21	-0.05
Metabolome	4-Hydroxybutyric acid		0.0000	0.0354	0.01	0.19
Metabolome	Leucinic acid		0.0000	-0.0342	-0.07	-0.42
Metabolome	LysoPI(18:0/0:0)		0.0000	0.0266	0.07	0.22
Metabolome	7 alpha-Hydroxy-3-oxo-4-cholestenoate		0.0000	-0.0227	-0.12	-0.40
Metabolome	PC(18:1(9Z)/18:2(9Z,12Z))		0.0000	0.0177	-0.29	0.41
Metabolome	PC(18:0/18:0)		0.0000	0.0076	0.07	0.23
Metabolome	2-hydroxy-3-methylvalerate		0.0000	-0.0042	-0.10	-0.35
Metabolome	3-Sialyl-N-acetyllactosamine		0.0000	0.0025	-0.13	-0.01
Metabolome	Uridine		0.0000	0.0011	0.20	0.12

Supplement Figure 2: Multiomic, integrative model feature associations based on component correlations can be interpreted within a circle plot with acute angles indicating positive correlations (red and green), obtuse angles representing negative correlations (purple), and right angles indicating no correlation (blue).

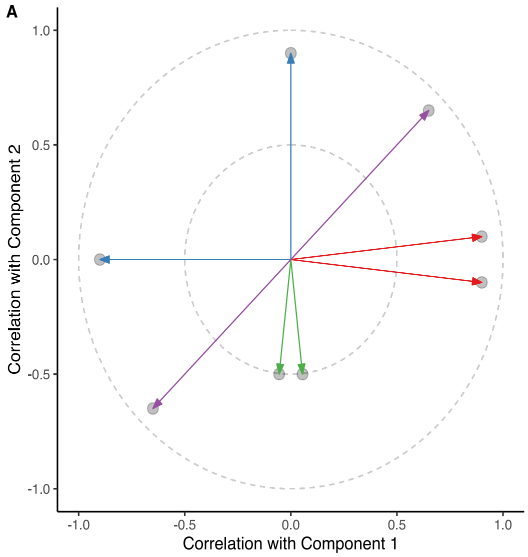

Supplement Table 6: Model selected metabolite features ordered by metabolite name, associated component, correlated clinical outcome, and metabolite class.

Metabolite Feature	Component	Clinical Outcome	Metabolite Class
Piperine	comp2	Healing	Alkaloids derived from ornithine
LysoPG(16:0/0:0)	comp2	Healing	Glycerophospholipids and Glycerolipids
MG(0:0/14:0/0:0)	comp2	Healing	Glycerophospholipids and Glycerolipids
MG(0:0/15:0/0:0)	comp2	Healing	Glycerophospholipids and Glycerolipids
MG(0:0/16:0/0:0)	comp2	Healing	Glycerophospholipids and Glycerolipids
MG(16:0/0:0/0:0)	comp2	Healing	Glycerophospholipids and Glycerolipids
MG(16:1(9Z)/0:0/0:0)	comp1	Healing	Glycerophospholipids and Glycerolipids
MG(16:1(9Z)/0:0/0:0)	comp2	Healing	Glycerophospholipids and Glycerolipids
PC(18:1(9Z)/18:2(9Z,12Z))	comp2	Healing	Glycerophospholipids and Glycerolipids
PC(18:2(9Z,12Z)/20:4(5Z,8Z,11Z,14Z))	comp2	Healing	Glycerophospholipids and Glycerolipids
PC(P-16:0/16:1(9Z))	comp2	Healing	Glycerophospholipids and Glycerolipids
N-Acetylasparagine	comp1	Healing	Alanine, aspartate and glutamate metabolism
Bicine	comp1	Healing	Amino Acid Derivatives, peptides, and organic acids
Cinnamoylglycine	comp1	Healing	Amino Acid Derivatives, peptides, and organic acids
Histidinyl-Alanine	comp2	Healing	Amino Acid Derivatives, peptides, and organic acids
Phenylalanyl-Alanine	comp2	Healing	Amino Acid Derivatives, peptides, and organic acids
Suberic acid	comp1	Healing	Amino Acid Derivatives, peptides, and organic acids
Valyl-Glutamine	comp1	Healing	Amino Acid Derivatives, peptides, and organic acids
N-Acetylcitrulline	comp2	Healing	Arginine and Proline
N-Acetylglutamic acid	comp2	Healing	Arginine and Proline

Methyl-4-hydroxybenzoate sulfate	comp2	Healing	Benzenoids
O-methoxycatechol-O-sulphate	comp1	Healing	Benzenoids
N-Acetyl-beta-alanine	comp1	Healing	Beta Alanine Metabolism
Taurochenodesoxycholic acid	comp1	Healing	Bile Acid Metabolism
Tetradecanedioic acid	comp1	Healing	Bile Acid Metabolism
Caffeine	comp1	Healing	Caffeine Metabolism
Capric acid	comp2	Healing	Caffeine Metabolism
Theophylline	comp1	Healing	Caffeine Metabolism
Cer(d17:1/16:0)	comp2	Healing	Ceramides
Cer(d18:1/14:0)	comp2	Healing	Ceramides
Cer(d18:1/24:2(5Z,9Z))	comp2	Healing	Ceramides
Cer(d18:2(4E,14Z)/16:0)	comp2	Healing	Ceramides
Cer(t18:0/16:0)	comp2	Healing	Ceramides
ceramide (d16:1/24:1, d18:1/22:1)	comp2	Healing	Ceramides
Ceramide (d18:1/16:0)	comp2	Healing	Ceramides
ceramide (d18:1/17:0, d17:1/18:0)	comp2	Healing	Ceramides
Ceramide (d18:1/18:0)	comp2	Healing	Ceramides
Ceramide (d18:1/20:0)	comp2	Healing	Ceramides
N-behenoyl-sphingadienine (d18:2/22:0)	comp2	Healing	Ceramides
2-iminopiperidine	comp1	Healing	Chemical
3-hydroxy-2-methylpyridine sulfate	comp1	Healing	Chemical
3-Hydroxypyridine sulfate	comp1	Healing	Chemical
Dimethylmalonic acid	comp1	Healing	Disaccharides and Oligosaccharides
De(carboxymethoxy) cetirizine acetic acid	comp1	Healing	Drug - Respiratory
Acetylglycine	comp1	Healing	Glycine, serine and threonine metabolism
Formiminoglutamic acid	comp1	Healing	Histidine Metabolism
1H-Indole-3-carboxaldehyde	comp1	Healing	Indoles and Derivatives
(R)-Pantoic acid	comp2	Healing	Lipids and Fatty Acids
10Z-Heptadecenoic acid	comp2	Healing	Lipids and Fatty Acids

13Z,16Z-docosadienoic acid	comp2	Healing	Lipids and Fatty Acids
2-Hydroxydecanedioic acid	comp1	Healing	Lipids and Fatty Acids
2-Hydroxydecanoate	comp1	Healing	Lipids and Fatty Acids
2-hydroxyheptanoate	comp2	Healing	Lipids and Fatty Acids
2-hydroxynervonate	comp2	Healing	Lipids and Fatty Acids
3-Hydroxycapric acid	comp1	Healing	Lipids and Fatty Acids
3-Hydroxyoctanoic acid	comp2	Healing	Lipids and Fatty Acids
8-Hydroxyoctanoate	comp1	Healing	Lipids and Fatty Acids
Adipic acid	comp1	Healing	Lipids and Fatty Acids
Azelaic acid	comp1	Healing	Lipids and Fatty Acids
Dodecanedioic acid	comp1	Healing	Lipids and Fatty Acids
Eicosenoic acid	comp2	Healing	Lipids and Fatty Acids
Erucic acid	comp2	Healing	Lipids and Fatty Acids
Hexadecanedioic acid	comp1	Healing	Lipids and Fatty Acids
Hydroxyoctanoic acid	comp1	Healing	Lipids and Fatty Acids
Hydroxyoctanoic acid	comp2	Healing	Lipids and Fatty Acids
Malonic acid	comp1	Healing	Lipids and Fatty Acids
Nonadeca-10(Z)-enoic acid	comp2	Healing	Lipids and Fatty Acids
octadecenedioate (C18:1-DC)	comp1	Healing	Lipids and Fatty Acids
Palmitoleic acid	comp2	Healing	Lipids and Fatty Acids
Pimelic acid	comp1	Healing	Lipids and Fatty Acids
Sebacic acid	comp1	Healing	Lipids and Fatty Acids
Undecanedioic acid	comp1	Healing	Lipids and Fatty Acids
2-Furoylglycine	comp1	Healing	Microbial Metabolite
Methylguanidine	comp1	Healing	Microbial Metabolite

Vanillin	comp1	Healing	Microbial Metabolite
Vanillin	comp2	Healing	Microbial Metabolite
N(2)-Acetyllysine	comp2	Healing	Nicotinate and nicotinamide metabolism
Trigonelline	comp1	Healing	Nicotinate and nicotinamide metabolism
glutamine conjugate of C6H10O2 (2)	comp1	Healing	Partially Characterized Molecules
glutamine conjugate of C7H12O2	comp1	Healing	Partially Characterized Molecules
2-dehydro-3-deoxy-D-gluconic acid	comp2	Healing	Pentose Phosphate Pathway
Quinic acid	comp1	Healing	Phenylalanine, tyrosine and tryptophan biosynthesis
2'-deoxyadenosine 3'-monophosphate	comp2	Healing	Purine and Pyrimidine Metabolism
Adenosine triphosphate	comp2	Healing	Purine and Pyrimidine Metabolism
hexadecasphingosine (d16:1)	comp2	Healing	Sphingolipid metabolism
sphingadienine	comp2	Healing	Sphingolipid metabolism
Sphingosine	comp2	Healing	Sphingolipid metabolism
7 alpha-Hydroxy-3-oxo-4-cholestenoate	comp2	Healing	Steroids and Sterols
pregnenetriol sulfate	comp1	Healing	Steroids and Sterols
Formylanthranilic acid	comp1	Healing	Tryptophan
Indole-3-carboxylic acid	comp1	Healing	Tryptophan
N-stearoyl-sphingadienine	comp2	Healing	Tryptophan
Tryptamine	comp2	Healing	Tryptophan
p-Cresol glucuronide	comp1	Healing	Tyrosine
2-Hydroxy-3-methylbutyric acid	comp2	Healing	Valine, leucine and isoleucine biosynthesis

2-hydroxy-3-methylvalerate	comp2	Healing	Valine, leucine and isoleucine biosynthesis
3-Hydroxyisovaleric acid	comp1	Healing	Valine, leucine and isoleucine biosynthesis
Leucinic acid	comp2	Healing	Valine, leucine and isoleucine biosynthesis
3-hydroxystachydrine	comp1	Non-Healing	Alkaloids derived from ornithine
Stachydrine	comp1	Non-Healing	Alkaloids derived from ornithine
LysoPE(20:4(5Z,8Z,11Z,14Z)/0:0)	comp1	Non-Healing	Glycerophospholipids and Glycerolipids
LysoPG(18:1(9Z)/0:0)	comp2	Non-Healing	Glycerophospholipids and Glycerolipids
LysoPG(18:2(9Z,12Z)/0:0)	comp2	Non-Healing	Glycerophospholipids and Glycerolipids
LysoPI(18:0/0:0)	comp2	Non-Healing	Glycerophospholipids and Glycerolipids
MG(16:3)	comp2	Non-Healing	Glycerophospholipids and Glycerolipids
PC(18:0/18:0)	comp2	Non-Healing	Glycerophospholipids and Glycerolipids
PC(18:2(9Z,12Z)/18:2(9Z,12Z))	comp2	Non-Healing	Glycerophospholipids and Glycerolipids
PC(P-16:0/16:0)	comp2	Non-Healing	Glycerophospholipids and Glycerolipids
PE(P-16:0/20:4(5Z,8Z,11Z,14Z))	comp1	Non-Healing	Glycerophospholipids and Glycerolipids
Allantoic acid	comp2	Non-Healing	Amino Acid Derivatives, peptides, and organic acids
Thioproline	comp2	Non-Healing	Amino Acid Derivatives, peptides, and organic acids
3-Sialyl-N-acetyllactosamine	comp2	Non-Healing	Amino Sugar Metabolism
N1,N8-Diacetylspermidine	comp1	Non-Healing	Arginine and Proline Metabolism
Caproic acid	comp2	Non-Healing	Caffeine Metabolism
Caprylic acid	comp2	Non-Healing	Caffeine Metabolism
MOPS	comp2	Non-Healing	Chemical
ApG	comp1	Non-Healing	Diribonucleotides

GpA	comp1	Non-Healing	Diribonucleotides
UpU	comp1	Non-Healing	Diribonucleotides
UpU	comp2	Non-Healing	Diribonucleotides
Sucrose	comp2	Non-Healing	Disaccharides and Oligosaccharides
Fibrinopeptide B (1-12)	comp1	Non-Healing	Fibrinogen Cleavage Peptide
4-Allylphenol sulfate	comp1	Non-Healing	Food Component/Plant
Acesulfame	comp1	Non-Healing	Food Component/Plant
Betonicine	comp1	Non-Healing	Food Component/Plant
Cysteineglutathione disulfide	comp1	Non-Healing	Glutathione Metabolism
D-Ribose	comp1	Non-Healing	Glycolysis / Gluconeogenesis and TCA and Pentose and Glucuronate and OXPHOS
D-Ribose	comp2	Non-Healing	Glycolysis / Gluconeogenesis and TCA and Pentose and Glucuronate and OXPHOS
fructose 1,6-diphosphate	comp1	Non-Healing	Glycolysis / Gluconeogenesis and TCA and Pentose and Glucuronate and OXPHOS
Pyruvic acid	comp1	Non-Healing	Glycolysis / Gluconeogenesis and TCA and Pentose and Glucuronate and OXPHOS
1-Methylhistamine	comp1	Non-Healing	Histidine Metabolism
3-Methylhistidine	comp1	Non-Healing	Histidine Metabolism
Histamine	comp1	Non-Healing	Histidine Metabolism
N-Acetyl-3-methylhistidine	comp1	Non-Healing	Histidine Metabolism

D-chiro-inositol	comp1	Non-Healing	Inositol phosphate metabolism
(2 or 3)-decenoate (10:1n7 or n8)	comp2	Non-Healing	Lipids and Fatty Acids
4-Deoxythreonic acid	comp1	Non-Healing	Lipids and Fatty Acids
4-Deoxythreonic acid	comp2	Non-Healing	Lipids and Fatty Acids
4-Hydroxybutyric acid	comp2	Non-Healing	Lipids and Fatty Acids
Butyric acid	comp2	Non-Healing	Lipids and Fatty Acids
DL-3-hydroxy caproic acid	comp2	Non-Healing	Lipids and Fatty Acids
Heptanoic acid	comp2	Non-Healing	Lipids and Fatty Acids
Pelargonic acid	comp2	Non-Healing	Lipids and Fatty Acids
5-(galactosylhydroxy)-L-lysine	comp1	Non-Healing	Lysine Metabolism
Aminoadipic acid	comp1	Non-Healing	Lysine Metabolism
N(6)-Methyllysine	comp1	Non-Healing	Lysine Metabolism
3-methyl pyruvic acid	comp2	Non-Healing	Methionine, Cysteine, SAM and Taurine Metabolism
N2-acetyl,N6,N6-dimethyllysine	comp2	Non-Healing	N-acyl-alpha amino acids
ADP-ribose	comp1	Non-Healing	Nicotinate and nicotinamide metabolism
ADP-ribose	comp2	Non-Healing	Nicotinate and nicotinamide metabolism
2',3' cyclic AMP	comp1	Non-Healing	Purine and Pyrimidine Metabolism
Adenosine	comp1	Non-Healing	Purine and Pyrimidine Metabolism
Guanosine	comp1	Non-Healing	Purine and Pyrimidine Metabolism
Guanosine diphosphate	comp1	Non-Healing	Purine and Pyrimidine Metabolism
Guanosine triphosphate	comp1	Non-Healing	Purine and Pyrimidine Metabolism

Uridine	comp2	Non-Healing	Purine and Pyrimidine Metabolism
Uridine triphosphate	comp1	Non-Healing	Purine and Pyrimidine Metabolism
2-Methylbutyroylcarnitine	comp1	Non-Healing	Valine, leucine and isoleucine biosynthesis
Alpha-Ketooctanoic acid	comp2	Non-Healing	Valine, leucine and isoleucine biosynthesis
N,N,N-trimethyl-5-aminovalerate	comp1	Non-Healing	Valine, leucine and isoleucine biosynthesis

Supplement Table 7: Annotated Metabolite Set Enrichment Analysis for Non-Healing Wounds Clinical Outcome.

Metabolic Pathway	Clinical Outcome	Enrichment Ratio
Gluconeogenesis	Non-healing	6.389776358
Purine Metabolism	Non-healing	3.623188406
Warburg Effect	Non-healing	3.703703704
Citric Acid Cycle	Non-healing	4.942339374
Fatty Acid Biosynthesis	Non-healing	4.518072289
Beta Oxidation of Very Long Chain Fatty Acids	Non-healing	6.211180124
Histidine Metabolism	Non-healing	3.768844221
Pyruvate Metabolism	Non-healing	3.367003367
Glycolysis	Non-healing	4.587155963
Methylhistidine Metabolism	Non-healing	13.19261214
Selenoamino Acid Metabolism	Non-healing	3.90625
Mitochondrial Beta-Oxidation of Short Chain Saturated Fatty Acids	Non-healing	3.90625
Pentose Phosphate Pathway	Non-healing	3.636363636
Starch and Sucrose Metabolism	Non-healing	3.401360544
Fructose and Mannose Degradation	Non-healing	3.401360544
Amino Sugar Metabolism	Non-healing	3.194888179
Aspartate Metabolism	Non-healing	3.012048193
Homocysteine Degradation	Non-healing	5.847953216
Galactose Metabolism	Non-healing	2.773925104
Pyruvaldehyde Degradation	Non-healing	5.263157895
Methionine Metabolism	Non-healing	2.512562814
Glucose-Alanine Cycle	Non-healing	4.048582996
Glutamate Metabolism	Non-healing	2.197802198
Alanine Metabolism	Non-healing	3.105590062
Pyrimidine Metabolism	Non-healing	1.851851852
Butyrate Metabolism	Non-healing	2.777777778
Lactose Synthesis	Non-healing	2.777777778
Glycine and Serine Metabolism	Non-healing	1.785714286
Nucleotide Sugars Metabolism	Non-healing	2.638522427
Threonine and 2-Oxobutanoate Degradation	Non-healing	2.638522427
Betaine Metabolism	Non-healing	2.512562814
Transfer of Acetyl Groups into Mitochondria	Non-healing	2.398081535

Cysteine Metabolism	Non-healing	2.028397566
Pterine Biosynthesis	Non-healing	1.883239171
Urea Cycle	Non-healing	1.883239171
Lysine Degradation	Non-healing	1.757469244
Ammonia Recycling	Non-healing	1.700680272
Beta-Alanine Metabolism	Non-healing	1.550387597
Nicotinate and Nicotinamide Metabolism	Non-healing	1.506024096
Propanoate Metabolism	Non-healing	1.256281407

Supplement Table 8: Annotated Metabolite Set Enrichment Analysis for Healing Wounds Clinical Outcome.

**Metabolic Pathway**	**Clinical Outcome**	**Enrichment Ratio**
Sphingolipid Metabolism	Healing	8.347245409
Beta Oxidation of Very Long Chain Fatty Acids	Healing	7.874015748
Caffeine Metabolism	Healing	5.813953488
Glycerolipid Metabolism	Healing	5.347593583
Tryptophan Metabolism	Healing	3.397508494
Aspartate Metabolism	Healing	3.816793893
Fatty Acid Biosynthesis	Healing	3.816793893
Biotin Metabolism	Healing	8.333333333
Thiamine Metabolism	Healing	7.407407407
Phenylacetate Metabolism	Healing	7.407407407
Lactose Degradation	Healing	7.407407407
Histidine Metabolism	Healing	3.179650238
Trehalose Degradation	Healing	6.060606061
Phosphatidylethanolamine Biosynthesis	Healing	5.555555556
Phosphatidylcholine Biosynthesis	Healing	4.761904762
Alanine Metabolism	Healing	3.937007874
Phosphatidylinositol Phosphate Metabolism	Healing	3.937007874
Spermidine and Spermine Biosynthesis	Healing	3.717472119
Butyrate Metabolism	Healing	3.521126761
Mitochondrial Electron Transport Chain	Healing	3.521126761
Lactose Synthesis	Healing	3.521126761
Ethanol Degradation	Healing	3.521126761
Bile Acid Biosynthesis	Healing	2.055498458
Nucleotide Sugars Metabolism	Healing	3.344481605
Glutathione Metabolism	Healing	3.344481605

Riboflavin Metabolism	Healing	3.344481605
Threonine and 2-Oxobutanoate Degradation	Healing	3.344481605
Pantothenate and CoA Biosynthesis	Healing	3.184713376
Betaine Metabolism	Healing	3.184713376
Sulfate/Sulfite Metabolism	Healing	3.039513678
Transfer of Acetyl Groups into Mitochondria	Healing	3.039513678
Glycolysis	Healing	2.906976744
Purine Metabolism	Healing	1.834862385
Inositol Phosphate Metabolism	Healing	2.78551532
Cysteine Metabolism	Healing	2.570694087
Oxidation of Branched Chain Fatty Acids	Healing	2.570694087
Phytanic Acid Peroxisomal Oxidation	Healing	2.570694087
Phenylalanine and Tyrosine Metabolism	Healing	2.475247525
Selenoamino Acid Metabolism	Healing	2.475247525
Mitochondrial Beta-Oxidation of Short Chain Saturated Fatty Acids	Healing	2.475247525
Mitochondrial Beta-Oxidation of Medium Chain Saturated Fatty Acids	Healing	2.475247525
Urea Cycle	Healing	2.386634845
Mitochondrial Beta-Oxidation of Long Chain Saturated Fatty Acids	Healing	2.386634845
Pentose Phosphate Pathway	Healing	2.304147465
Folate Metabolism	Healing	2.304147465
Inositol Metabolism	Healing	2.227171492
Ammonia Recycling	Healing	2.155172414
Starch and Sucrose Metabolism	Healing	2.155172414
Fructose and Mannose Degradation	Healing	2.155172414
Citric Acid Cycle	Healing	2.087682672
Amino Sugar Metabolism	Healing	2.024291498
Gluconeogenesis	Healing	2.024291498
Nicotinate and Nicotinamide Metabolism	Healing	1.908396947
Galactose Metabolism	Healing	1.757469244
Propanoate Metabolism	Healing	1.589825119
Methionine Metabolism	Healing	1.589825119
Fatty acid Metabolism	Healing	1.552795031
Pyruvate Metabolism	Healing	1.420454545
Steroid Biosynthesis	Healing	1.390820584
Glutamate Metabolism	Healing	1.390820584
Arginine and Proline Metabolism	Healing	1.285347044
Pyrimidine Metabolism	Healing	1.172332943

Warburg Effect	Healing	1.172332943
Glycine and Serine Metabolism	Healing	1.132502831
Valine, Leucine and Isoleucine Degradation	Healing	1.132502831

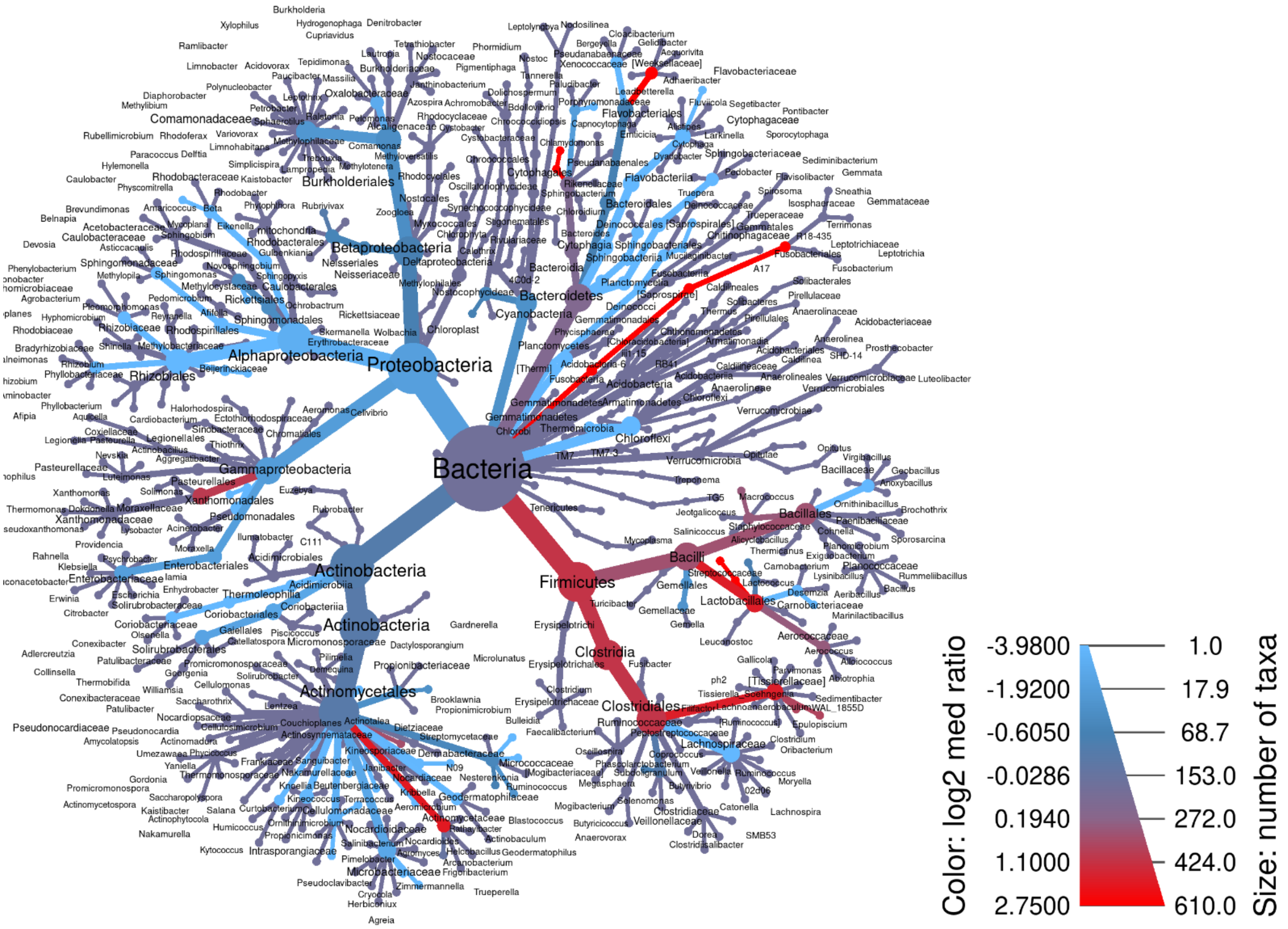

Supplement Figure 3: *Heattree showing the relative abundance and phylogenetic relationships of the 635 taxa derived from 16S rRNA analysis.* These were the taxa that were input into the initial model. The color indicates the Log2 ratio of median proportions of taxa, those that are more abundant in non-responders (color more Red) and those more abundant in Responders (colors more Blue).
